# Narazaciclib, a novel multi-kinase inhibitor with potent activity against CSF1R, FLT3 and CDK6, shows strong anti-AML activity in defined preclinical models

**DOI:** 10.1038/s41598-024-59650-y

**Published:** 2024-04-19

**Authors:** Tao Yang, Hang Ke, Jinping Liu, Xiaoyu An, Jia Xue, Jinying Ning, Feng Hao, Lingxin Xiong, Cen Chen, Yueying Wang, Jia Zheng, Bing Gao, Zhengzheng Bao, Kefeng Gong, Lei Zhang, Faming Zhang, Sheng Guo, Qi-Xiang Li

**Affiliations:** 1 Hanx Biopharmaceuticals, Ltd., Wuhan, Hubei, PRC China; 2 Crown Bioscience, Inc., Taicang, Jiangsu, PRC USA; 3 Kyinno Biotechnology, Ltd., Beijing, PRC China

**Keywords:** HX301/ON123300, RTK, TKi, Biomarker, LSC, PDX, Cancer, Drug discovery, Oncology

## Abstract

CSF1R is a receptor tyrosine kinase responsible for the growth/survival/polarization of macrophages and overexpressed in some AML patients. We hypothesized that a novel multi-kinase inhibitor (TKi), narazaciclib (HX301/ON123300), with high potency against CSF1R (IC_50_ ~ 0.285 nM), would have anti-AML effects. We tested this by confirming HX301’s high potency against CSF1R (IC_50_ ~ 0.285 nM), as well as other kinases, *e.g*. FLT3 (IC_50_ of ~ 19.77 nM) and CDK6 (0.53 nM). An in vitro proliferation assay showed that narazaciclib has a high growth inhibitory effect in cell cultures where CSF1R or mutant FLT3-ITD variants that may be proliferation drivers, including primary macrophages (IC_50_ of 72.5 nM) and a subset of AML lines (IC_50_ < 1.5 μM). In vivo pharmacology modeling of narazaciclib using five AML xenografts resulted in: inhibition of MV4-11 (FLT3-ITD) subcutaneous tumor growth and complete suppression of AM7577-PDX (FLT3-ITD/CSF1R^med^) systemic growth, likely due to the suppression of FLT3-ITD activity; complete suppression of AM8096-PDX (CSF1R^hi^/wild-type FLT3) growth, likely due to the inhibition of CSF1R (“a putative driver”); and nonresponse of both AM5512-PDX and AM7407-PDX (wild-type FLT3/CSF1R^lo^). Significant leukemia load reductions in bone marrow, where disease originated, were also achieved in both responders (AM7577/AM8096), implicating that HX301 might be a potentially more effective therapy than those only affecting peripheral leukemic cells. Altogether, narazaciclib can potentially be a candidate treatment for a subset of AML with CSF1R^hi^ and/or mutant FLT3-ITD variants, particularly second generation FLT3 inhibitor resistant variants.

## Introduction

Acute myeloid leukemia (AML) is an aggressive leukemia of myeloid lineage with different subtypes and diverse molecular pathology. It has the highest mortality rate among hematological malignances, with a global annual mortality of ~ 150,000. The molecular pathogenic heterogeneity of AML results in varying outcomes to the current treatment regimens. The first-line treatment is usually induction chemotherapy (*e.g*., cytarabine), which results in remission in many cases but is not curative. The relapse rate is approximately 33–78%, most likely due to residual disease, particularly reserved leukemia-initiation cells (LICs) in bone marrow ^1^. The next-line treatments are further chemo-/radiation, or stem cell transplantation, etc. Targeted therapy tailored for specific genetic or epigenetic driver lesions could be alternative treatment options for defined subsets of patients, e.g., patients with IDH1/2 mutations^2^ and patients with FLT3-ITD or FLT-TKD mutations^1,3^. All these treatments, although being effective for specific subsets of patients, still mostly led to relapse similarly due to residual diseases or emergence of resistance mutations. Therefore, AML still remains an unmet medical need.

FLT3 is a class III receptor tyrosine kinase (RTK) expressed on hematopoietic stem cells (HSCs)^4^, as well as leukemic blast cells and leukemogenic stem cells (LSCs). Autoactivation of FLT3 independent of its ligand (FL) interaction is usually associated with internal tandem duplications (ITDs) within the juxtamembrane domain or point mutations in the kinase domain activation loop (TKD: tyrosine kinase domain) and has been demonstrated to be one of the key leukemogenic drivers found in one-third of AML patients. FLT3-ITD and TKD mutants are also poorer prognosis markers of AML diseases^5^ and have been explored as effective therapeutic targets for patients with such mutations^6^. Several FLT3 inhibitors, including gilteritinib, quizartinib (AC220) and midostaurin^7^, have been approved for the treatment of FLT3-ITD^+^ and/or TKD^+^ AML patients via using an FDA-approved test. Because of the rapid development of drug resistance, new drugs with high potency against resistant mutant AML are becoming urgent unmet medical needs calling for next generation FLT3 inhibitors.

**Figure 1:**
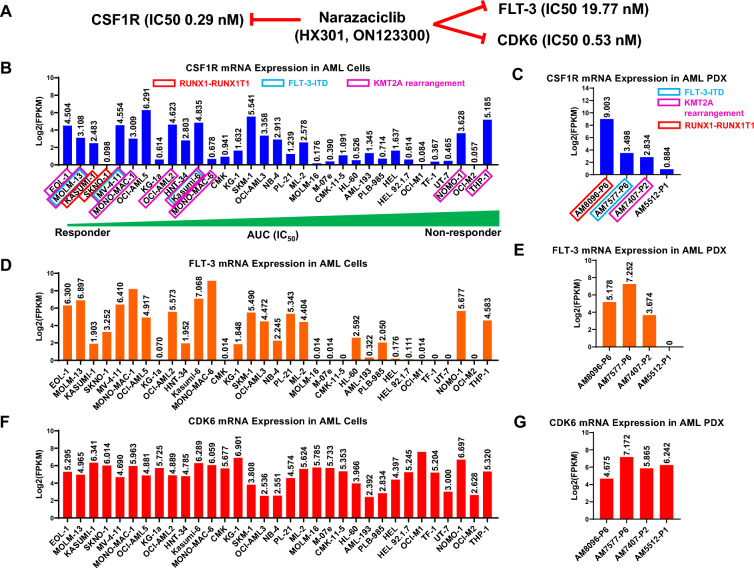
CSF1R, FLT3 and CDK6 mRNA expression levels in AML cell lines and patient-derived xenografts (PDXs). (**A**) Differential potency of narazaciclib against multiple kinases. (**B**) CSF1R mRNA expression in 33 AML cell lines showing declining response to narazaciclib from left to right, with FLT-3-ITD mutation in MV-4-11, Kasumi-1, and MOLM-13 cells as well as KMT2A-MLLT3 fusion gene in MV-4-11, MONO-MAC-6, NOMO-1 and THP-1 cells; (**C**) CSF1R mRNA expression in 4 AML-PDXs, with FLT3-ITD mutation in AM7577; (**D**) FLT3 mRNA expression in 33 AML cell lines; (**E**) FLT3 mRNA expression in 4 AML-PDXs; (**F**) CDK6 mRNA expression in 33 AML lines; (**G**) CDK6 mRNA expression in 4 AML-PDXs. mRNA expression levels are shown as Log2 (FPKM, Fragments Per Kilobase Million). AML cell lines or AML-PDX bearing FLT3-ITD mutation were highlighted in blue box, while AML cell lines or AML-PDX carrying KMT2A-MLLT3 fusion were highlighted in purple box.

In addition to genetic or cytogenetic lesions, some epigenetic changes have also been associated with AMLs. CDK6, not CDK4, has also been found to be overexpressed in many AML and associated with poor prognosis^8–10^. CDK6 overexpression was also believed to be part of the two leukemogenic driving pathways downstream of MLL-translocation (a well-known leukemogenic driver lesion^11–13^) and FLT3-ITD^9,10,14^. Hypothetically, CDK6 inhibitors (CDK6i) could be another potential drug target approach for managing AML (Fig. 1A), potentially synergizing with upstream FLT3i, or be effective on its own for treating FLT3i-resistant patients (Fig. 1A).

CSF1R, or colony-stimulating factor 1 receptor, also called M-CSF-R (macrophage colony-stimulating factor receptor), is an RTK and belongs to the same family as FLT3. CSF1R is responsible for the growth, survival and polarization of certain myeloid lineages of cells, including monocytes and macrophages. High CSF1R expression seems to be a biomarker of cancer prognosis^15^, and it has been considered to be a potential anticancer drug target. In addition, AML patients with inversion of chromosome 16 [inv(16)(p13q22)] which results in the fusion transcript *CBFb-MYH11* showed upregulated expression of CSF1R and disruption of the CSF1R signaling via a CSF1R inhibitor significantly reduced inv(16) AML tumor growth^16^. Moreover, a recent study revealed that blocking of CSF1R signaling inhibited AML tumor growth by disrupting paracrine signals from supporting cells^17^. Several clinically investigational CSF1R inhibitors (CSF1Ri), either TKIs or antibodies^18–20^, are being developed, but with limited success, except for tenosynovial giant cell tumors (TGCT), where CSF1R alteration is the sole driver of the disease^21^. Since AML is a malignancy of myeloid lineage, we hypothesize that the aberrant CSF1R expression could be a putative leukemogenic driver in certain subsets of AML patients, *e.g*. inv (16) AML, which can be targeted by CSF1Ri.

Narazaciclib (HX301 or ON123300) is an investigational multi-kinase inhibitor (TKi) being developed for treating different cancers due to its broad potency against CSF1R, FLT3, CDK4/6, ARK5, etc. It demonstrated robust antitumor activities in experimental cancer models in vitro and in vivo presumably due to one or more the above-mentioned potencies in these model systems^22–25^. We hypothesized that narazaciclib should have the desired antileukemic activity in a subset of AMLs, particularly where CSF1R and FLT3 (including FLT3-ITD/TKD) are leukemic drivers (Fig. 1A). This report revealed that a variety of experimental AML models, including AML cell lines and patient-derived xenograft (PDX), with defined genetic/cytogenetic/epigenetic alterations, are indeed sensitive to narazaciclib. Together, as a potent kinase inhibitor for CSF1R, FLT3 and CDK6, narazaciclib could potentially become a novel treatment for these subsets of AMLs.

## Results

### CSF1R, along with FTL3, MLL and CDK6, seems uniquely overexpressed in some AML patients, patient-derived models and immortalized cell lines

Since M-CSF (CSF-1)/CSF1R signaling axis plays vital roles in the survival, growth and differentiation of the cells of myeloid lineages, it is reasonable to expect that its genetic/epigenetic aberrations may possibly play critical roles in certain myeloid malignances, similar to those reported for FLT3-ITD/TKD mutations, MLL fusion and CDK6 overexpression^8–10,12–14^. To this end, we first examined the publicly available AML data (*gepia.cancer-pku.cn*) and found that the CSF1R gene is overexpressed in many AML patient samples (Supplement Fig. S1A), similar to several other AML-implicated targets previously, FLT3 (Supplement Fig. S1B), MLL (Supplement Fig. S1C) and CDK6 (Supplement Fig. S1D). A recent report also confirmed that CSF-1 and CSF1R are upregulated in AML patients with *CBFb-MYH11* fusion gene. Therefore, it is possible that the overexpression or overactivations of CSF1R, could potentially be involved in the pathogenesis of this subset of AMLs, similar to the above three known leukemogenic drivers^8–10,12–14^. CSF1R could in theory be a potential drug target for AML. Additionally, the report that CSF1R was only expressed in AML LSCs (leukemic stem cells) but not in HSCs (normal hematopoietic stem cells) further implicated CSF1R as an interesting AML target with less on-target off-leukemia toxicity^26^.

Adequate CSF1R-expressing AML experimental systems could be important to test the above hypothesis. We therefore performed RNAseq on a panel of 33 immortalized AML cell lines to examine CSF1R gene expression. As shown in Fig. 1B, CSF1R is differentially expressed in these AML cell lines. Those cell lines with high CSF1R expression could thus potentially be tested for the role of CSF1R in AML. FLT3, MLL and CDK6 overexpression was also similarly found in some of the AML cell lines (Fig. 1D,F, respectively), likely relevant to their respective driver properties. It was worth noting that while mRNA levels of these genes were measured, their protein levels as well as their phosphorylation states have not been investigated in this study. mRNA levels are not always correlated to their protein levels.

Since the overexpression of CDK6 expression has been suggested as the result of FLT3-ITD and MLL translocation^8–10,12–14^, we were thus keen on examining the correlations among the expression of these four putative leukemogenic genes. Pairwise correlations for these four genes across 173 AML patients from TCGA were analyzed, and the data are summarized in Supplemental Fig. S1G. The results showed positive correlations between CDK6 and MLL, and between CDK6 and FLT3 expression (to a lesser degree, also between FLT3 and MLL), which is consistent with the previous reports^8–10,12–14^. Interestingly, the results also showed a negative correlation between CSF1R and CDK6 expression but no correlation between CSF1R and FLT3 or between CSF1R and MLL expression. This might imply that the putative CSF1R-driven AML has independent molecular pathogenesis different from those of FLT3- and MLL-driven AML.

Patient-derived xenografts (PDXs) have been considered closely mimicking patient pathology and predicting drug response^18–20^. Engraftment of AML patient bone marrow cells to immunodeficient mice to create an AML-PDX model, AM7577 (M5 subtype with FLT3-ITD mutation and IDH2 R140Q mutation), is suitable for pharmacological evaluation of new AML targeted drugs, e.g., FLT3/FLT3-ITD inhibitor (FLT3i)^1^ and IDH2-mutant inhibitor (IDH2^mut^i)^2^. Other three validated AML-PDX models, AM8096 (M2 subtype with RUNX1-RUNX1T1 fusion and CEBPA mutation), AM5512 (M7 subtype) and AM7407 (M4 subtype with KMT2A rearrangement) (Supplement Table S1) are also similarly suitable for pharmacology modeling. Altogether, all four models could stably be passed serially in animals and develop full-blown leukemia with 100% mortality. These models were characterized for leukemia growth kinetics and/or response to two standard of care (SOC) drugs for AML, including Cytarabine and Azacytidine, as shown in Supplement Fig. S2 or reported previously^1^. CSF1R gene expression in all four AML PDXs was also determined by RNAseq. As shown in Fig. 1C, CSF1R is highly expressed in AM8096 AML-PDX, followed by AM7577, AM7407 and AM5512, supporting the above hypothesis that CSF1R could be a potential drug target for a subset of AML with high CSF1R expression.

### Genetic lesions found in AML experimental models

In addition to epigenetics, there are two potential genetic lesions of interest in this study, FLT3-ITD and MLL (KMT2A)-rearrangement, both of which are well known to be leukemogenic drivers^1,9,11,12,21^, with FDA-approved drugs on the market for targeting FLT3/FLT3-ITD and for targeting MLL-partner protein (Menin)^22–24^. We therefore examined the FLT3-ITD status and KMT2A rearrangement among the experimental models mentioned above based on both RNAseq and whole exome sequencing (WES) datasets. As shown in Fig. 1B, three AML cell lines including MV-4-11, MOLM-13 and Kasumi-6, and one AML-PDX, AM7577, carry FLT3-ITD mutations^1^, whereas the remaining 3 AML-PDX models including AM8096, AM7407 and AM5512 have wild-type FLT3. Additionally, 9 AML cell lines including EOL-1, MOLM13, MV-4-11, MONO-MAC-1, MONO-MAC-6, OCI-AML-2, Kasumi-6, NOMO-1 and THP-1, have KMT2A rearrangement, and AM7407 AML-PDX has KMT2A rearrangement as well. Moreover, two cell lines, Kasumi-1 and SKNO-1 bear RUNX1-RUNX1T1 fusion. RUNX1 (Runt-related transcription factor 1, or acute myeloid leukemia 1 protein (AML1)) and RUNX1T1 gene fusion is also frequently found in AML^25^. Furthermore, we also found one recurrent CSF1R missense mutation, e.g., CSF1R^V279M^, in two AML cell lines, Kasumi-6 and NOMO1, and another CSF1R missense mutation, CSF1R^G413S^, in the HL-60 cell line. Therefore, these genetically defined AML cell lines and AML-PDX with epigenetic expression profiles would allow us to peek into the biological significance of CSF1R blocking and possibly the cross-talk between CSF1R and other known genetic alterations in AML.

### Narazaciclib specifically inhibited the in vitro proliferation of CSF1R-driven recombinant Ba/F3 cell lines and primary M2 macrophage

**Figure 2:**
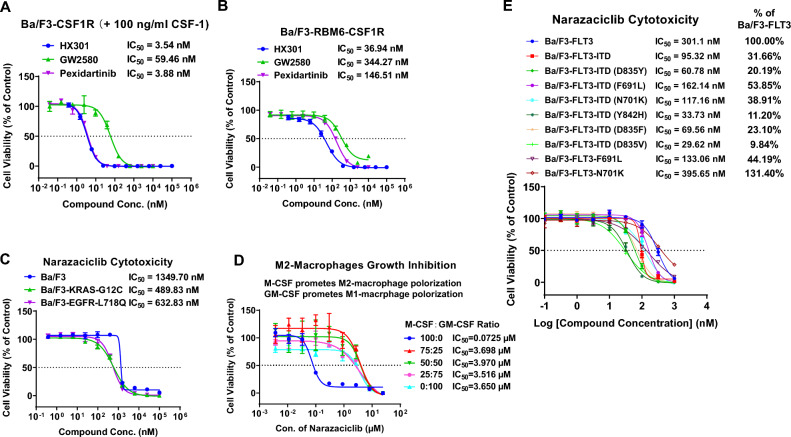
In vitro cell viability assay of narazaciclib in different cell culture systems. (**A**) Inhibition of cell proliferation by narazaciclib in Ba/F3-CSF1R cell line; (**B**) Inhibition of cell proliferation by narazaciclib in Ba/F3-RBM6-CSF1R cell line; (**C**) Inhibition of proliferation by narazaciclib in Ba/F3-KRAS-G12C, Ba/F3-EGFR-L718Q, Ba/F3 cell lines; (**D**) Inhibition of M-CSF/CSF1R induced M2 primary macrophage growth by narazaciclib. (**E**) In vitro cell viability assay of narazaciclib in various FLT3-ITD/TKD cell lines **and** comparison of the potency of narazaciclib between wild-type FLT3 and FLT3-ITD/TKD cell lines.

To investigate whether CSF1R is of relevance for AML, we utilized an investigational tyrosine kinase inhibitor (TKi) in clinic trials, narazaciclib (HX301 or ON123300), which possesses strong inhibitory potency against CSF1R. As shown in Fig. 1 and Supplemental Fig. S3, narazaciclib demonstrated preferential inhibition on the enzymatic activity of CSF1R (IC_50_ ~ 0.285 nM), in comparison to another two kinase, CDK6 (0.53 nM) and FLT3 (IC_50_ ~ 19.77 nM). In addition, narazaciclib was reported to inhibit several other kinases of oncological interest, *e.g*., CDK4, ARK5, PIK3-δ, and FGFR1^26^.With the demonstrated CSF1R enzymatic inhibition by narazaciclib (Supplement Fig. S3A), we next set out to test whether CSF1R inhibition by narazaciclib is relevant for the cell proliferation of CSF1R^+^ cells. Ba/F3 cells are a mIL3 (murine interleukin-3)-dependent pro-B cell line whose proliferation can be dependent on some activated protein kinases in the absence of IL-3. Antagonizing such kinase-dependent cell growth by corresponding kinase inhibitors can thus be used to determine the impact of specific kinase inhibitors on cell proliferation. First, we tested narazaciclib in the two CSF1R-recombinant mouse Ba/F3 cell lines where CSF1R is overexpressed and functions as a driver for cell proliferation, for the Ba/F3-CSF1R line in the presence of CSF1 (M-CSF) (Fig. 2A) and for Ba/F3-RBM6-CSF1R not requiring CSF1 (Fig. 2B). As shown in Fig. 2A, narazaciclib potently inhibited the proliferation of Ba/F3-CSF1R cells in the presence of CSF1 with an IC_50_ of ~ 3.54 nM, similar to a FDA-approved CSF1R inhibitor (CSF1Ri) for treating Tenosynovial giant cell tumors (TGCT), pexidartinib^27^ (or PLX-3397, IC_50_ ~ 3.88 nM) but 16 times more potent than another CSF1Ri, GW2580, with an IC_50_ of 59.46 nM. Narazaciclib, along with these two CSF1Ri, also showed significant growth inhibition in the Ba/F3-RBM6-CSF1R line (overexpressing RBM6-CSF1R fusion protein), with IC_50_ values of 36.94, 344.27 and 146.51 nM, respectively (Fig. 2B). Furthermore, narazaciclib showed little inhibitory activity against the parental Ba/F3 line (IC_50_ of 1349.70 nM) and the two recombinant Ba/F3 cells with unrelated drivers, KRAS^G12C^ (IC_50_ of 489.83 nM) and EGFR^L718Q^ (IC_50_ of 632.83 nM) (Fig. 2C). Altogether, these data suggested that the potent inhibition of the proliferation of those CSF1R-driven Ba/F3 recombinant lines (Ba/F3-CSF1R and Ba/F3-RBM6-CSF1R) is solely due to the specific inhibition of CSF1R by Narazaciclib.

Since the well-known physiological function of CSF1R is to support the survival, growth, differentiation and polarization of macrophages, we also performed a proliferation assay in primary macrophage culture. A high ratio of M-CSF to GM-CSF triggers macrophages to polarize toward M2 phenotype, which upregulates CSF1R expression. As shown in Fig. 2D, narazaciclib clearly demonstrated potent inhibition of M2-macrophage proliferation with an IC_50_ of 72.5 nM, once again confirming its specific suppression of CSF1R biological signaling in another relevant cell-based assay.

### Narazaciclib inhibited the proliferation of the recombinant Ba/F3-FLT3 mutant variant cell lines more potently than wild-type line

With the demonstrated FLT3 enzymatic inhibition by narazaciclib (Supplement Fig. S3B), we next set out to test whether narazaciclib have additive inhibitory activities against the proliferation of cells driven by FLT3-ITD/TKD mutations. As shown in Fig. 2E, while narazaciclib caused some degree of inhibition on the proliferation of recombinant wild-type Ba/F3-FLT3 cell (IC_50_ 301.10 nM), it showed a stronger inhibition on the proliferation of recombinant Ba/F3-FLT3-ITD cell line (IC_50_ 95.32 nM).

Although the second generation FLT3 inhibitors, such as quizartinib (AC220) and gilteritinib, demonstrated enhanced potency against FLT3 in comparison to the first generation FLT3 inhibitors (*e.g*., sorafenib, sunitinib, etc.) in clinical trials, relapse still occurs quickly (within several months) after initial remission due to emergence of drug resistance. FLT3-tyrosine kinase domain (TKD) mutations in the activation loop (*e.g.,* D835Y) or the gate-keeper site (*e.g.,* F691L) have been identified to be one of the causes for the FLT3-drug resistance. As shown in Fig. 2E, narazaciclib exhibited stronger inhibition on the proliferation of recombinant Ba/F3-FLT3-ITD-F691L cells (IC_50_ 162.14 nM), Ba/F3-FLT3-F691L cells (IC_50_ 133.06 nM), Ba/F3-FLT3-ITD-D835Y cells (60.78 nM), Ba/F3-FLT3-ITD-D835F cells (69.56 nM), Ba/F3-FLT3-ITD-D835V cells (29.62 nM), Ba/F3-FLT3-ITD-Y842H cells (33.73 nM), Ba/F3-FLT3-ITD-N701K cells (117.16 nM), respectively, in comparison to the wild type Ba/F3-FLT3 cells. Taken together, these results demonstrated that narazaciclib possesses preferential potency in inhibiting recombinant Ba/F3 cell lines with disease driven- and resistant- FLT3-ITD or FLT3-TKD mutations over wild type FLT3. Since FLT3 is also present on normal blood cells, an inhibitor of such would potentially has less on-target toxicity thus wider therapeutic window. Moreover, this additive inhibition of FLT3-ITD/TKD besides CSF1R would broaden its therapeutic application for AML.

### Narazaciclib potently inhibited the proliferation of a subset of AML cell lines, including those with FLT3-ITD

Given the confirmed TKi activity of narazaciclib against CSF1R, FLT3 and CDK6 above and its impact on the proliferation of CSF1R- or FLT3-ITD/TKD-driven recombinant cells, we next tested the anti-AML activity of narazaciclib. First, we screened a panel of 33 immortalized and genetically defined AML cell lines (Supplemental Table S2) using in vitro proliferation assays. As shown in Fig. 1, Table 1 and Supplemental Fig. S4, their sensitivities to narazaciclib vary greatly and can be presented by the area under the dose–response curve (AUC) as an alternative to IC_50_ (for details, see Supplemental Fig. S5). If we arbitrarily select a cutoff AUC < 2.4 as sensitive cell lines to narazaciclib, there are 15 out of 33 sensitive cell lines with AUCs from 0.59 to 2.37 (EOL1, MOLM13, KASUMI-1, MV-4-11, OCI-AML-5, MONO-MAC-1, SKNO1, OCI-AML-2,, KASUMI-6,KG1A, HNT34, MONO-MAC-6, CMK, SKM-1, and PL-21). As expected, all 4/4 (100%) FLT3-ITD-carrying cell lines, MOLM-13, KASUMI-6, MV-4-11 and PL-21 (carrying both FLT3-ITD and wild type FLT3), are sensitive to narazaciclib. This result confirmed that FLT3-ITD is the leukemogenic driver in these three cell lines and the hypothesis that FLT3-ITD AML is sensitive to narazaciclib. Interestingly, these four AML cell lines also seem to have relatively higher CDK6 expression, implicating CDK6 could be a putative leukemogenic driver as documented in other reports^10,14^. Therefore, we cannot rule out the anti-CDK6 role in the contribution of the anti-AML activity of narazaciclib in these four FLT3-ITD AML cell lines. Nevertheless, the putative dual targeting of FLT3-ITD and CDK6 by narazaciclib would be even more advantageous over FLT3-single-targeting drugs for FLT3i-resistant patients.

**Table 1:** Narazaciclib IC_50_ in 33 AML cell lines in the in vitro proliferation assay.

Cell line	IC_50_ (μM)	AUC	Classification by AUC	Note
EOL1	0.035	0.59174876	Sensitive	
MOLM-13	0.103	1.06845352	Sensitive	FLT3-ITD
KASUMI-1	0.169	1.30705327	Sensitive	
SKNO-1	0.234	1.97041677	Sensitive	
MV-4–11	0.264	1.45355673	Sensitive	FLT3-ITD
MONOMAC-1	0.297	1.9058795	Sensitive	
OCI-AML-5	0.370	1.63648209	Sensitive	
KG-1A	1.056	2.28943281	Sensitive	
OCI-AML-2	1.156	2.11623796	Sensitive	
HNT34	1.477	2.0682284	Sensitive	
KASUMI-6	1.482	2.2511886	Sensitive	FLT3-ITD
MONOMAC-6	1.520	2.35618105	Sensitive	
CMK	1.649	2.34683758	Sensitive	
KG-1	1.761	2.62977977	Intermediate	
SKM-1	1.937	2.32307747	Sensitive	
OCI-AML-3	2.940	2.86283471	Insensitive	
NB4	3.282	2.86496327	Insensitive	
PL21	3.402	2.36955672	Sensitive	WT FLT-3 and FLT3-ITD
ML2	3.604	2.6331517	Intermediate	
MOLM-16	4.772	2.99198803	Insensitive	
M07E	4.861	2.55742129	Intermediate	
CMK-115	5.070	2.80067574	Insensitive	
HL-60	6.138	2.83603296	Insensitive	
AML-193	6.294	2.51414275	Intermediate	
PLB-985	14.090	2.98805625	Insensitive	
HEL	15.280	3.33477637	Insensitive	
HEL-9217	15.717	3.20864348	Insensitive	
OCI-M1	19.489	3.40402505	Insensitive	
TF-1	50.757	3.50234816	Insensitive	
UT7	62.372	3.46940769	Insensitive	
NOMO-1	> 100	3.2071439	Insensitive	
OCI-M2	> 100	3.46042133	Insensitive	
THP-1	> 100	3.44468973	Insensitive	

AUC ≤ 2.4, sensitive; 2.4 < AUC < 2.8, intermediate; AUC ≥ 2.8, insensitive.

Aside from these four FLT3-ITD AML cell lines, there are another 11 sensitive cell lines with wild-type FLT3. 7 out of them expressed relatively high levels of CSF1R, except SKNO1, CMK, PL-21 and KG1A (Fig. 1B). It is possible that the robust anti-AML activity of narazaciclib (AUC < 2.4) for EOL-1, KASUMI-1, MONO-MAC-1, OCI-AML-5, OCI-AML-2, and SKM-1 cells might be attributed to the inhibition of CSF1R. In addition, among these 11 non-FLT3-ITD AML cell lines, 4 cell lines including EOL-1, MONO-MAC-1, OCI-AML-2 and MONO-MAC-6 carry KMT2A (MLL) rearrangement (Supplemental Table S2). In fact, these AML cell lines with KMT2A (MLL) rearrangement also express high levels of CDK6, as shown in Fig. 1F. Taken together, these results imply that the anti-AML activity of narazaciclib in these cell lines might be mediated by CSF1R and CDK6 dual targeting. For SKNO1/KG1A/CMK cell lines with neither FLT3-ITD nor high-level expression of CSF1R or CDK6, the MOA of narazaciclib sensitivity is still unknown and needs further investigation.

### In vivo pharmacological evaluation of narazaciclib in AML xenograft models

**Figure 3:**
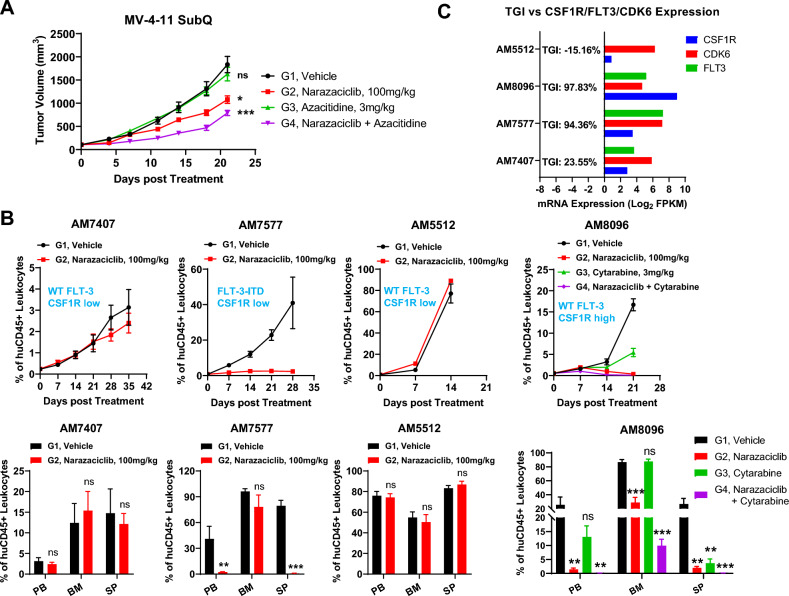
In vivo anti-AML activity of narazaciclib in preclinical AML xenograft models. (**A**) narazaciclib inhibited MV-4-11 tumor growth and outperformed Azacitidine in a subcutaneous AML xenograft model. (**B**) narazaciclib showed anti-AML effects on AM7577 and AM8096 AML PDX models but displayed little anti-AML effects on AM7407 and AM5512 AML PDX models. (**C**) Comparison of tumor growth inhibition (TGI) among 4 AML-PDX models with differential CSF1R, FLT3 and CDK6 expression levels as well as genetic characteristics. In the MV-4-11 subcutaneous xenograft, narazaciclib was intraperitoneally administrated at 100 mg/kg twice per week. In the AM7407, AM7577 and AM5512 AML PDX models, narazaciclib was orally administrated at 100 mg/kg every day, whereas in the AM8096 AML PDX model narazaciclib was intraperitoneally administrated at 100 mg/kg every day. Graphs in A and B showed mean tumor volume ± standard error of the mean (SEM). Significance was calculated using two-way ANOVA and one-way ANOVA with post-hoc comparisons between treatment groups and vehicle group. *, *p* < 0.05; **, *p* < 0.01; ***, *p* < 0.001.

With the confirmed in vitro anti-AML activity in AML cell lines with either FLT3-ITD mutation, or high CSF1R expression, or high CDK6 expression, we decided to test the anti-AML activity of narazaciclib in vivo. In a FLT3-ITD^+^ MV-4-11 subcutaneous xenograft model, as shown in Fig. 3A, one of the standards of care (SOC) treatments for AML, Azacitidine, showed little anti-AML effect. In contrast, narazaciclib at 100 mg/kg demonstrated significant anti-AML activity. Moreover, combination of narazaciclib and Azacitidine further improved the anti-AML effects on MV-4–11 tumor growth. This result was consistent with prior knowledge that MV-4-11 responded to FLT3i, such as sorafenib, a nonspecific TKi with anti-FLT3i activity^21^, and quizartinib (AC220) and gilteritinib, two more specific and approved FLT3i^1,28^. Therefore, FLT3-ITD is the leukemogenic driver for this model, and the observed in vivo anti-AML activity of narazaciclib in this model likely resulted from its suppression of FLT3-ITD kinase activity^30^.

Next, to further confirm the anti-leukemic activity of narazaciclib with more confident prediction of patient pharmacology, we chose to test narazaciclib in systemic AML-PDX models^1^. The pharmacology results are shown in Fig. 3B. Firstly, in AM7577 AML-PDX model bearing FLT3-ITD mutation, which is known to be an oncogenic driver of this model as described previously^1^, narazaciclib at 100 mg/kg completely diminished the leukemic load in peripheral blood. This strong anti-AML activity is thus likely due to the drug’s suppression of FLT3-ITD. Second, AM8096, with wild-type FLT3 and extremely high expression levels of CSF1R (Fig. 1C), also responded completely to narazaciclib. In contrast, AM8096 responded poorly to quizartinib (Supplement Fig. S6), suggesting that wild type FLT3 is not a leukemogenic driver in this model and narazaciclib might provide advantageous anti-AML effects over quizartinib for patients with wild type FLT3. In addition, there was no expression of CSF1 in this model (https://www.crownbio.com/database), excluding the possibility of the CSF1-autocrine disease mechanism. It is thus highly possible that the overexpression of CSF1R is the driver for AM8096 leukemogenesis. Third, AM5512 and AM7407, both with wild-type FLT3 and little CSF1R expression (Fig. 1C), did not respond to narazaciclib at all, suggesting that neither FLT3 nor CSF1R has a role in leukemogenesis in these two models (Fig. 3B). Altogether, it seems again that either FLT-ITD+ and/or CSF1R highly-expressing AML respond to narazaciclib, while others do not.

In addition, the careful examination of narazaciclib’s effect in those two responding AML-PDX models (AM7577 and AM8096) showed that narazaciclib not only reduce the leukemic load in peripheral blood and spleen, but also the leukemic load in bone marrow, in sharp contrast to that of AraC (Fig. 3B). We described a similar phenomenon previously observed for quizartinib (AC220)^1^, where we proposed that AC220 affected leukemic stem cells (LSCs), particularly in bone marrow, and caused long-term remission and even cure^1^. It is highly possible that narazaciclib may cause a similar impact on LSCs and have long-lasting AML inhibition or even cure where CSF1R is the disease driver. This putative scenario is also consistent with the report that CSF1R was overexpressed in LSCs but not in HSCs^29^. Furthermore, it is worth noting that combination with azacytidine can even further enhance the anti-AML activity (Fig. 3B).

**Figure 4:**
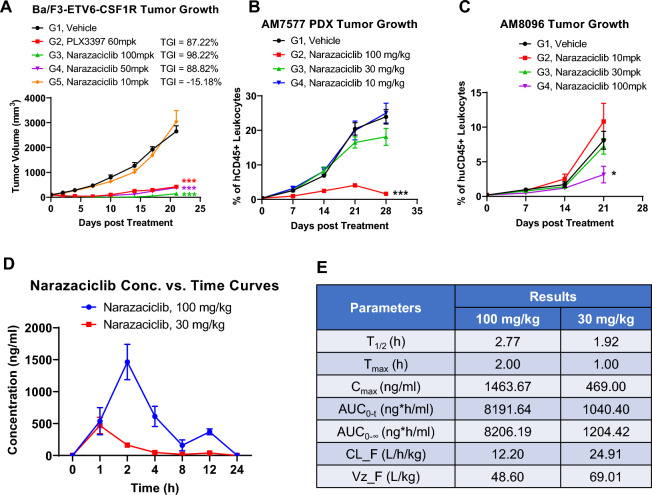
Pharmacodynamics versus pharmacokinetics of Narazaciclib in mouse models. Panel A/B/C demonstrate that Narazaciclib inhibited AML tumor growth in a dose-dependent manner: (**A**) inhibition of the tumor growth of a CSF1R driven Ba/F3-ETV6-CSF1R xenograft in immunodeficient mice; (**B**) reduction of the tumor burden of a FLT3-ITD-bearing AM7577 AML-PDX; C. anti-AML activities in a CSF1R highly expressing AM8096 AML-PDX (Tumor growth curves are shown as mean tumor volume ± standard error of the mean (SEM). Significance was calculated using two-way ANOVA with post-hoc comparisons between treatment groups and vehicle group. *, *p* < 0.05; **, *p* < 0.01; ***, *p* < 0.001). Panel D/E: Pharmacokinetic (PK) evaluation of narazaciclib in non-tumor bearing mice**.** (**D**) The plasma concentration-curves of narazaciclib following a single oral administration at 100 mg/kg or 30 mg/kg. (**E**) PK parameters after the single-dose administration of narazaciclib at 100 mg/kg or 30 mg/kg orally. T_1/2_, elimination half-life; T_max_, Time to peak drug concentration; C_max_, maximum concentration; AUC_0-∞_, area under the plasma concentration–time curve from zero to infinity; AUC_0-t_, area under the plasma concentration–time curve from zero to the time of the last quantifiable time-point; CL_F, total body clearance; Vz-F, total volume of distribution. All data are presented as mean tumor volume ± standard error of the mean (SEM) of three animals per group.

To further explore the anti-AML activity of narazaciclib via targeting FLT3/FLT3-ITD or CSF1R, we tested different dose levels of narazaciclib in a CSF1R driven Ba/F3-ETV6-CSF1R xenograft model along with those two AML-PDX models (AM7577 and AM8096) responding well to narazaciclib. As shown in Fig. 4A, narazaciclib at either 50 mg/kg or 100 mg/kg significantly delayed the tumor growth, similar to an approved CSF1Ri, PLX3397. In the AM7577 and AM8096 AML-PDX models, narazaciclib reduced the leukemic burdens in a dose-dependent manner, (Fig. 4B,C), where narazaciclib at 100 mg/kg demonstrated the maximal anti-AML effects in vivo. Moreover, narazaciclib at 100 mg/kg also significantly reduced the leukemic burdens in both spleen and bone marrow in the AM7577 AML-PDX model (Supplemental Fig. S7). Altogether, it is possible that narazaciclib could have anti-AML activity through MOAs targeting either FLT3/FLT3-ITD or possibly CSF1R, depending on the genetic/epigenetic compositions of AML patients.

### Potential predictive factors for the narazaciclib response

Per the above in vitro and in vivo trials on the cohort of preclinical AML models thus far, FLT3-ITD seems to be one of the positive predictors for the response to narazaciclib in AML patients (Figs. 1B, 3, 4 and Supplemental Fig. S8 Right panel). This is quite consistent with the employment of FLT3-ITD companion diagnostic (CDx) assay to select AML patients with FLT3-ITD mutation in clinic, which are more likely to respond to FLT3 inhibitors. We could thus believe that the same CDx can be used for guiding the clinical development of narazaciclib.

In light of the known putative targets of narazaciclib and their overexpression in many AML patients (Supplement Fig. S1), AML cell lines and AML-PDX models (Fig. 1B–G), we were keen to ask if the expression levels of these putative targets are predictive of the response to narazaciclib. Therefore, we performed statistical analysis on the expression levels of CSF1R, CDK6, FLT3 and KMT2A (MLL) between sensitive AML cell lines to narazaciclib and insensitive AML cell lines (Fig. 1B–G and Table 1). As shown in Supplemental Fig. S8 left panel. the results revealed that CSF1R and FLT3 both are upregulated in sensitive AML cell lines, whereas CDK6 and KMT2A did not show any statistically significant difference in expression levels between those two subgroups. In conclusion, these results suggest that additional CDx based on the expression level of CSF1R and FLT3 might be used to select AML patients for narazaciclib in clinics.

### Preliminary pharmacokinetic analysis of the oral administration of narazaciclib and its effective anti-AML exposure in mice

In order to understand the effective anti-AML exposure of narazaciclib in mice, we conducted a preliminary pharmacokinetic (PK) analysis of a single-dose oral administration of narazaciclib, at the same dose levels as in anti-AML pharmacology study above, in non-tumor bearing mice. Following a single oral administration of narazaciclib at either 30 mg/kg or 100 mg/kg in mice, plasma samples were collected at various timepoints. The plasma concentrations of narazaciclib were analyzed by LC–MS. The concentration–time profiles of narazaciclib in plasma and the PK parameters of narazaciclib are summarized in Fig. 4E. The concentration of narazaciclib increased rapidly with a T_max_ of 2 h and a C_max_ of 1463.67 ng/ml for narazaciclib at 100 mg/kg, or a T_max_ of 1 h and a C_max_ of 469.00 ng/ml for narazaciclib at 30 mg/kg, respectively. In addition, narazaciclib undergoes relatively fast elimination from plasma with a T_1/2_ of 2.77 h for narazaciclib at 100 mg/kg or 1.92 h at 30 mg/kg. From these results we have preliminarily determined that the efficacious exposures of narazaciclib for anti-AML in mice is below 8191.64 ng*h/mL in terms of AUC_0~∞_ or 1463.67 ng/ml in terms of C_max_, corresponding to its 100 mg/kg dose level in mice, which can be used to guide clinical test to ensure the efficacious exposure in patients and the corresponding dose levels.

## Discussion

Our preclinical data seems to justify a further clinical testing of narazaciclib to treat AMLs with FLT3-ITD and/or FLT3i-resistant FLT3-TKD mutations due to its preferential inhibition on FLT3-TKD mutant variants over wild type FLT3. Nevertheless, the less potent activity against wild-type FTL3 can usually resulted in less on-target toxicity and increase the therapeutic window. The approved FLT3i for treating AML requires the use of companion diagnostics (CDx), e.g., the LeukoStrat CDx FLT3 Mutation Assay, to select patients with this specific mutation. Likely, the clinical development of narazaciclib for FLT3-ITD^+^ AML patients would also require a similar CDx as well as additional kits to detect resistant FLT3-TKD mutants. CDK6 overexpression was shown to be caused by FLT3-ITD and plays a role in FLT3-ITD-driven AML, which makes it targetable by CDK6i for AML treatment^10,14^. To this end, narazaciclib could be particularly effective for treating FLT3-ITD resistant mutant variants since it targets both FLT3-ITD and CDK6 in the same signaling pathway. Therefore, it could particularly be more advantageous for treating FLT3i-resistant AML. Indeed, there is a report that a small molecule, AMG925, with both FLT3 and CDK4/6 inhibitory activities has been identified for strong anti-AML, particularly anti-AML LSC^30^. It would be interesting to compare the profiles between narazaciclib and AMG925.

Our report suggests that CSF1R is a putative leukemogenic driver in some subsets of AMLs (Fig. 3 and Supplemental Fig. S8) and is thus targetable for the treatment of these AML patients, which is consistent with suggestions by others^17^. Our data demonstrated that AML-PDX models, without FLT3-ITD but with CSF1R as a putative leukemogenic driver (*e.g.*, possibly overexpression), responded to narazaciclib. In particular, CSF1R was reported to be expressed in LSCs but not HSCs^29^, suggesting less toxicity of narazaciclib on normal hematopoiesis. Furthermore, additional targeting on LSCs in bone marrow by narazaciclib, as shown in Fig. 3 and Supplemental Fig. S7, could be even more important for longer-term remission.

Unlike FLT3-ITD patients where a single genetic biomarker (FLT3-ITD) defines responders, the exact patient populations where CSF1R is the leukemogenic driver remained to be determined. Firstly, CSF1R mutations seemed not to be the biomarkers defining the main patient populations where CSF1R is the leukemic driver. Secondly, although CSF1R mRNA expression level seems to correlate with the response to narazaciclib in a selection of AML cell lines (Fig. 1B and Supplemental Fig. S8) and AML-PDX models (Fig. 3C), a few AML cell lines (OCI-AML-3, NOMO-1 and THP-1) with relatively high CSF1R expression showed suboptimal response to narazaciclib. Therefore, identification of additional epigenetic biomarkers (expression level of a panel of genes) as well as corresponding CDx are important for future clinical development of narazaciclib. There may be additional advantages for narazaciclib as it can potentially treat two subgroups of patients, driven by either CSF1R overexpression or FLT3-ITD, so it has broader applications than other known FLT3i. Besides, if there was a subset of AML patients with both CSF1R overexpression and FLT3-ITD, narazaciclib will be even more powerful than FLT3i alone.

Chromosomal rearrangement (11q23) results in H3K4 methyltransferase mixed-lineage leukemia (MLL) gene fusion, impacting gene expression of the stem cell program and driving leukemia-initiating activity. MLL represents 5–10% of primary AML and aggressive subtypes of leukemia with poorer prognosis and no readily targeted strategy^11,12^. CDK6 overexpression has been reported to be associated with MLL translocation and plays a role in AML pathogenesis and thus has been proposed to be an alternative target for MLL-driven AML diseases^9,12,13^. In our AML cell line panel, 7 out of 9 AML cell lines with MLL-rearrangement showed increased CDK6 expression as well as good response to narazaciclib (Fig. 1B,F). Moreover, KMT2A rearrangement and RUNX1-RUNX1T1 gene fusion are associated with high expression level of the lineage TF myocyte enhancer factor (MEF2C), which is a marker of poor prognostics in AML patients (Supplemental Fig. S1F) and controls AML cell proliferation^31,32^. Salt-inducible kinase 3 (SIK-3) is also highly expressed in AML patients (Supplemental Fig. S1E) and has been shown to maintain the function of MEF2C by phosphorylating HDAC in AML^31,32^. SIK-3 or MEF2C genetical deletion or inhibitors significantly suppressed the proliferation of MEF2C highly-expressing AML cells^32^. Remarkably, narazaciclib also demonstrated potent inhibition on SIK-3 along with its two family members, SIK-1 and SIK-2 (Supplemental Table S4). It’s worth noting that the good response of AML cell lines with KMT2A rearrangement to narazaciclib might be a result of the SIK-3 inhibition by narazaciclib, although this additional anti-AML effect of narazaciclib via targeting SIK-3 remains further investigation. Therefore, KMT2A rearrangement and its associated upregulation of CDK6 expression and/or MEF2C expression might also be predictive biomarkers for narazaciclib in AML.

From our data, narazaciclib impacted bone marrow significantly more than induction chemotherapy in model AM8096, where there is no FLT3-ITD, as similarly seen for quizartinib in AM7577^1^ (Fig. 3B). Bone marrow is the location where most LSCs reside and leukemia originates. Our data thus implicated the impact of narazaciclib on LSCs and may have the possibility of causing long-term remission, similar to FLT3i, where induction chemotherapy lacks, although our study have not specifically investigated narazaciclib’s effect on LSCs In other words, narazaciclib could become another potential targeted therapy for a new subset of AML, offering longer-term remission by preventing relapse, similar to FLT3i^1^. Our preclinical data also further demonstrated that the narazaciclib combination treatment together with induction SOC has additional activity in models driven by both FLT3-ITD (MV4-11) and CSF1R (AM8096) (Fig. 3B), similar to FLT3i shown in the preclinical model^1^. Therefore, a similar clinical strategy could be explored for narazaciclib.

Finally, the coupled efficacy and PK studies reported here enable us to determine the adequate anti-AML efficacious exposures, which can be translated into putative clinical efficacious exposure. This would help guide desired clinical dose to ensure this adequate exposure. Narazaciclib is currently under early-stage clinical evaluation (NCT05731934), and it can potentially be developed as a novel AML treatment.

## Materials and methods

### Cell lines and animals

All the AML cell lines used in in vitro assays were provided by the following vendors: Crown Bioscience (San Diego, USA), KYinno Biotechnology Co., Ltd. (Beijing, China), and Shanghai ChemPartner Co., Ltd. (Shanghai, China). All in vivo murine experiments were conducted under specific-pathogen-free (SPF) conditions at Crown Bioscience animal facility and KYinno Biotechnology animal facility in strict accordance with the Guide for the Care and Use of Laboratory Animals of the National Institutes of Health. The protocol and any amendments or procedures were reviewed and approved by the Committee on the Ethics of Animal Experiments of Crown Bioscience (Crown Bioscience IACUC Committee) or the Committee on the Ethics of Animal Experiments of KYinno Biotechnology (KYinno Biotechnology IACUC Committee) prior to execution. The study design all followed the ARRIVE Guideline and all the studies were conducted in accordance with the regulations of the Association for Assessment and Accreditation of Laboratory Animal Care (AAALAC). The antitumor treatment studies were conducted by following protocols described in detail elsewhere ^19,33^ with certain modifications.

### Kinase inhibition assay

Different assays were used to measure the inhibitory potencies of narazaciclib on FLT3, CSF1R and CDK6. For FLT3 inhibition, the enzyme, substrate (final concentration at 15 μg/μL), ATP (final concentration at 10 μM) and inhibitors were diluted and mixed in kinase buffer (40 mM Tris, pH 7.5; 20 mM MgCl_2_; 0.1 mg/ml BSA; 50 μM DTT). The mixtures were incubated at 25 °C for 120 min. Then, ADP‐Glo™ Reagent (Promega) was added to the mix and incubated at 25 °C for 40 min followed by the addition of kinase detection reagent and another 30 min at 25 °C. Luminescence was recorded with an integration time of 0.5 s.

For CSF1R inhibition, TK-substrate-biotin and ATP were incubated at room temperature for 40 min, followed by adding different concentrations of XL 665-labeled Narazaciclib and TK-antibody-Cryptate into each well of the assay plate and incubation at room temperature for 1 h. The fluorescence signal was read at 615 nm (Cryptate) and 665 nm (XL665) on an Envision 2104 plate reader.

For CDK6 inhibition, a Time-resolved fluorescence energy transfer (TR-FRET) kinase assay was performed according to the manufacturer’s instructions. Briefly, narazaciclib at various concentrations was pre-incubated with CDK6-CyclinD1 in kinase buffer at 25 °C for 1 h and then incubated with 12.5 nM Ulight-4E-BP1 peptide and 250 µM ATP at 25 °C for 2 h. The reaction was stopped by incubating with EDTA-containing stop buffer at 25 °C for 5 min. Subsequently, the mixture was incubated with detection buffer at 25 °C for 1 h. Finally, the signal was read on a Nivo Reader in TR-FRET mode (excitation at 320 nm and emission at 665 nm and 615 nm). The signal of the blank control was subtracted from the maximum signal (DMSO without compound) as well as from the value of narazaciclib at each concentration. This corrected emission ratio (ER) was used to calculate the percentage of enzyme activity recovery: enzyme activity recovery (%) = [(narazaciclib ER – blank control ER)-(max control ER-blank control ER)] × 100. The results were fitted to a dose–response curve by a 4-parameter log-logistic model:

$$Inhibition\%={\text{Bottom}}+\frac{{\text{Top}}-\mathrm{ Bottom}}{1+{10}^{\left({{\text{logEC}}}_{50}-x\right)*{\text{HillSlope}}}}$$

### Ba/F3 and primary macrophage proliferation assays

The recombinant Ba/F3 cell lines overexpressing human CSF1R, or human RBM6-CSF1R, or human ETV6-CSF1R were generated using a retrovirus vector (Kyinno Co., Ltd. Beijing). The recombinant Ba/F3 lines overexpressing human Kras G12C orEGFR-L718Q mutants, and different mutant variants of FLT3 were also similarly created as well. A standard proliferation assay via CTG readout was performed according to the vendor’s instructions (KYinno Biotechnology Co., Ltd. Beijing).

Human primary monocytes were isolated with an EasySep™ monocyte isolation kit (Stem Cell) from cryopreserved human PBMCs. Macrophages were differentiated and polarized to M1- or M2-macrophages from cultured monocytes in vitro in the presence of different concentration ratios of M-CSF and GM-CSF (100:0, 75:25, 50:50, and 0:100 ng/ml). Narazaciclib was serially diluted with culture medium to 9 final concentrations from 0.004 µM to 24 µM. The differentiated macrophages were incubated for 7 days at 37 °C followed by incubation with Cell Counting-Lite2.0 reagent (Vazyme) for 5 min on a shaker. Luminescence was recorded at 700 nm wavelength. The proliferation inhibition IC_50_ (half maximal inhibition concentration) of narazaciclib was determined by fitting the dose–response data to a four-parameter stimulation model.

### Proliferation assay of AML cell lines

Narazaciclib was serially diluted with culture medium to 9 final assay concentrations from 0.01 to 100 μM. The assay was performed with both a negative control (vehicle) and a positive control (Cisplatin) in triplicate. The AML cell lines at log-growth phase were cultured in 96-well plates (black transparent flat bottom 96-well plate, Corning, Cat# 3603, FBS, ExCell Bio., Cat# FND500) in the presence of narazaciclib or Cisplatin at various concentrations for 72 h or 120 h (only for AML-193 cell line), followed by CTG readout according to the manufacturer’s instruction (CellTiter-Glo Luminescent Cell Viability Assay, Promega, Cat# G7573).

$${\text{Viability }}\left( \% \right) = \left( {{\text{Lum}}_{{{\text{drug}}}} - {\text{Lum}}_{{\text{medium control}}} } \right)/\left( {{\text{Lum}}_{{\text{cell control}}} - {\text{Lum}}_{{\text{medium control}}} } \right) \times {1}00\% .$$

$${\text{Lum}}_{{\text{cell control}}} - {\text{Lum}}_{{\text{medium control}}} \;{\text{as}}\;{1}00\% ,{\text{ LumMediumcontrol }}\;{\text{as}}\; \, 0\% .$$

$${\text{Proliferation }}\left( {\text{fold increase}} \right) \, = \, \left( {{\text{D5}} - {\text{Lum}}_{{\text{None treated}}} - {\text{Lum}}_{{\text{Medium control}}} } \right) \, /\left( {{\text{D2Lum}}_{{\text{None treated}}} - {\text{Lum}}_{{\text{Medium control}}} } \right)$$

Dose–response curves were fitted by a 4-parameter log-logistic model:

$$Inhibition\%={\text{Bottom}}+\frac{{\text{Top}}-\mathrm{ Bottom}}{1+{10}^{\left({{\text{logEC}}}_{50}-x\right)*{\text{HillSlope}}}}$$

where Top and Bottom are the two asymptotes of the sigmoidal curve, EC_50_ is the relative IC_50_, concentration x is in log-10 scale and HillSlope is the slope at the EC_50_ point on the fitted curve. The fitted area under the curve (AUC) was calculated by:

$$Inhibition\%={\text{Bottom}}+\frac{{\text{Top}}-\mathrm{ Bottom}}{1+{10}^{\left({{\text{logEC}}}_{50}-x\right)*{\text{HillSlope}}}}$$

where a represents the log10-transformed minimal concentration and b represents the log10-transformed maximal concentration. An extremely strong positive correlation between the absolute IC_50_ and AUC is shown in Supplemental Fig. S5. To avoid NA values, AUC instead of IC_50_ was used as the in vitro efficacy endpoint to define sensitive (AUC < 2.40) and insensitive (AUC > 2.80) cell lines.

### In vivo pharmacology

The subcutaneous cell line xenograft (CDX) models and systemic patient-derived xenograft (PDX) models of AML have been extensively described before^31^. For the subcutaneous MV-4-11 CDX model, 5 × 10^6^ MV-4-11 tumor cells harvested from exponential growth phase were mixed with Matrigel (1:1) and then inoculated into the right front flank region of 6- to 7-week-old female NOD/scid mice for tumor development. MV-4-11 tumor-bearing mice were randomized into 5 groups (N = 8) when their tumor volume reached approximately 100 mm^3^ and received their designated treatments following randomization. Narazaciclib was administrated intraperitoneally at 100 mg/kg twice per week for 3 weeks, while Azacitidine was administrated intraperitoneally at 3 mg/kg twice per week for 3 weeks. For the subcutaneous Ba/F3-ETV6-CSF1R CDX model, 1 × 10^6^ Ba/F3-ETV6-CSF1R tumor cells harvested from exponential growth phase were mixed with Matrigel (1:1) and then inoculated into the right front flank region of 6- to 8-week-old female NOD/scid mice for tumor development. Ba/F3-ETV6-CSF1R tumor-bearing mice were randomized into 5 groups (N = 6) when their tumor volume reached approximately 100 mm^3^ and received their designated treatments following randomization. Narazaciclib was administrated orally at 10 mg/kg, or 50 mg/kg, or 100 mg/kg every day for 21 days, while PLX3397 (Pexidartinib) was administrated orally at 60 mg/kg every other day for 21 days. Tumor volume and body weight were measured at different time points and tumor growth inhibition (TGI) was used as index to determine the therapeutic efficacy.

For systemic AML-PDX models, the model establishment and narazaciclib pharmacology experiment were performed essentially as described previously^1^. Briefly, 2 × 10^6^ AM7407 cells, or 2 × 10^6^ AM7577 cells, or 2 × 10^6^ AM5512 cells, or 1.65 × 10^6^ AM8096 cells were inoculated intravenously into 4- to 5-week-old female NOD/scid mice for AML development. Engraftment was confirmed by the presence of circulating human CD45+ cells (anti-human CD45 antibody, Cat. 304038, Biolegend) in peripheral blood. Mice with successful engraftment of AML-PDX were randomized into different treatment groups (N = 6) and received their designated treatments. Narazaciclib was administrated orally or intraperitoneally at 10 mg/kg, or 30 mg/kg, or 100 mg/kg every day throughout the study, while Cytarabine was administrated intraperitoneally at 3 mg/kg every day. Following the initiation of designated treatments, leukemic burden was monitored by flow cytometry with anti-human CD45 antibody on the peripheral blood every week as well as on the peripheral blood, bone marrow cells and splenocytes of mice at sacrifice. Mice were sacrifice once the followings are observed: body weight loss over 20%, reduced motility and activity, labored breathing, ruffled coat, hunched back and severe dehydration.

### Pharmacokinetic (PK) analysis

Noncompartmental analyses (NCA) were performed using WinNonlinTM Phoenix, version XXX software (Pharsight, Cary, NC) to estimate the PK parameters including T_1/2_, elimination half-life; T_max_, Time to peak drug concentration; C_max_, maximum concentration; AUC_0-∞_, area under the plasma concentration–time curve from zero to infinity; AUC_0-t_, area under the plasma concentration–time curve from zero to the time of the last quantifiable time-point; CL_F, total body clearance; Vz-F, total volume of distribution. Following a single oral administration of narazaciclib at either 100 mg/kg or 30 mg/kg in female NOD/scid mice (N = 3), plasma samples were collected from 3 mice at each timepoint (pre-dose, 1h, 2h, 4h, 8h, 12h and24hpost administration) and analyzed by LC–MS.

### Supplementary Information

Supplementary Figures.

Supplementary Tables.

## Data Availability

The datasets used and/or analyzed during the current study available from the corresponding author on reasonable request.
