# Supplementary material for: Narazaciclib, a novel multi-kinase inhibitor with potent activity against CSF1R, FLT3 and CDK6, shows strong anti-AML activity in defined preclinical models
Source: Sci Rep. 2024 Apr 19;14:9032. doi: 10.1038/s41598-024-59650-y (PMC11031590; doi:10.1038/s41598-024-59650-y)
Supplement: Supplementary file 1 — Supplementary Figures. [file 41598_2024_59650_MOESM1_ESM.pptx]

## Slide 1
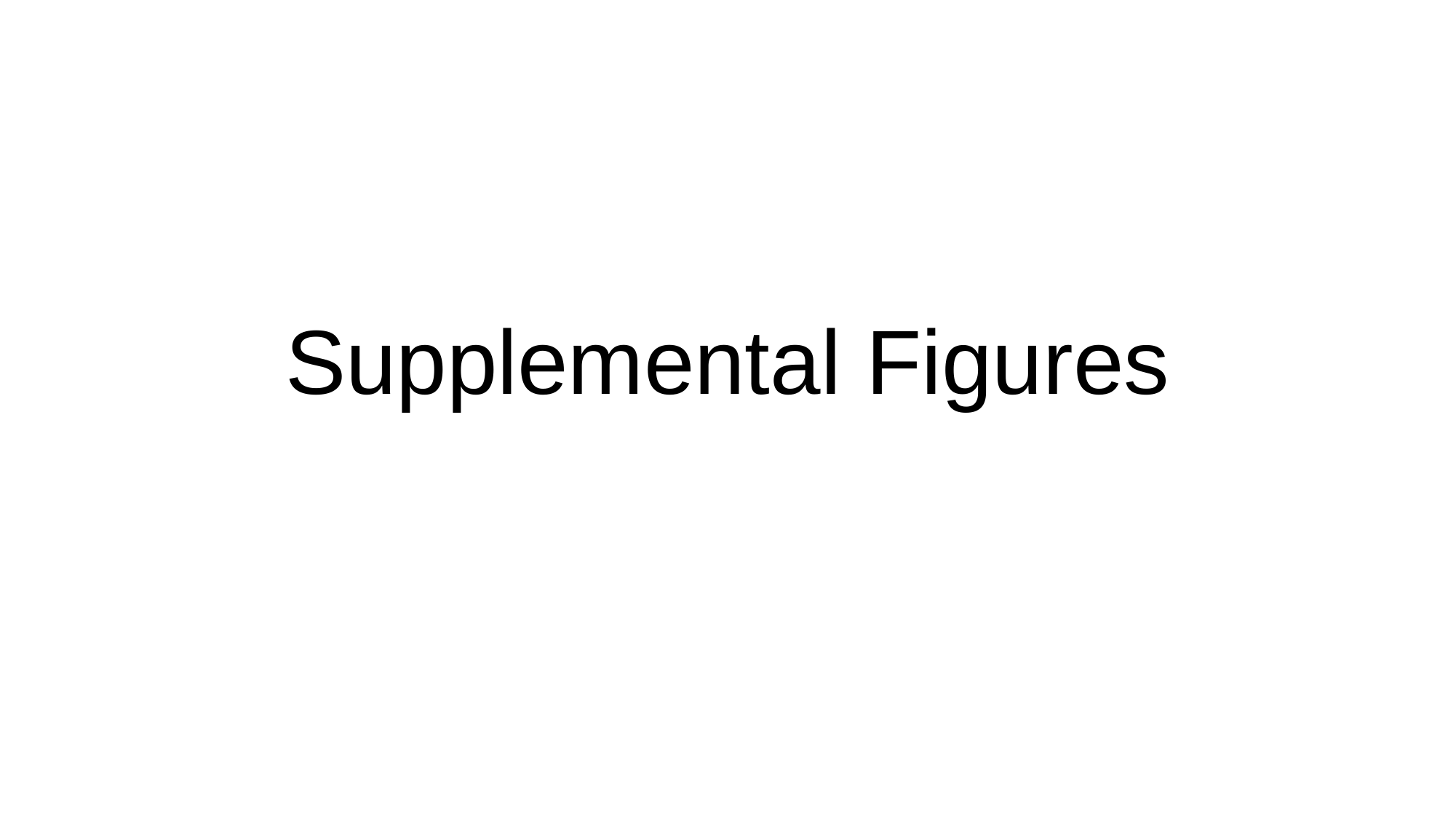

# Supplemental Figures

## Slide 2
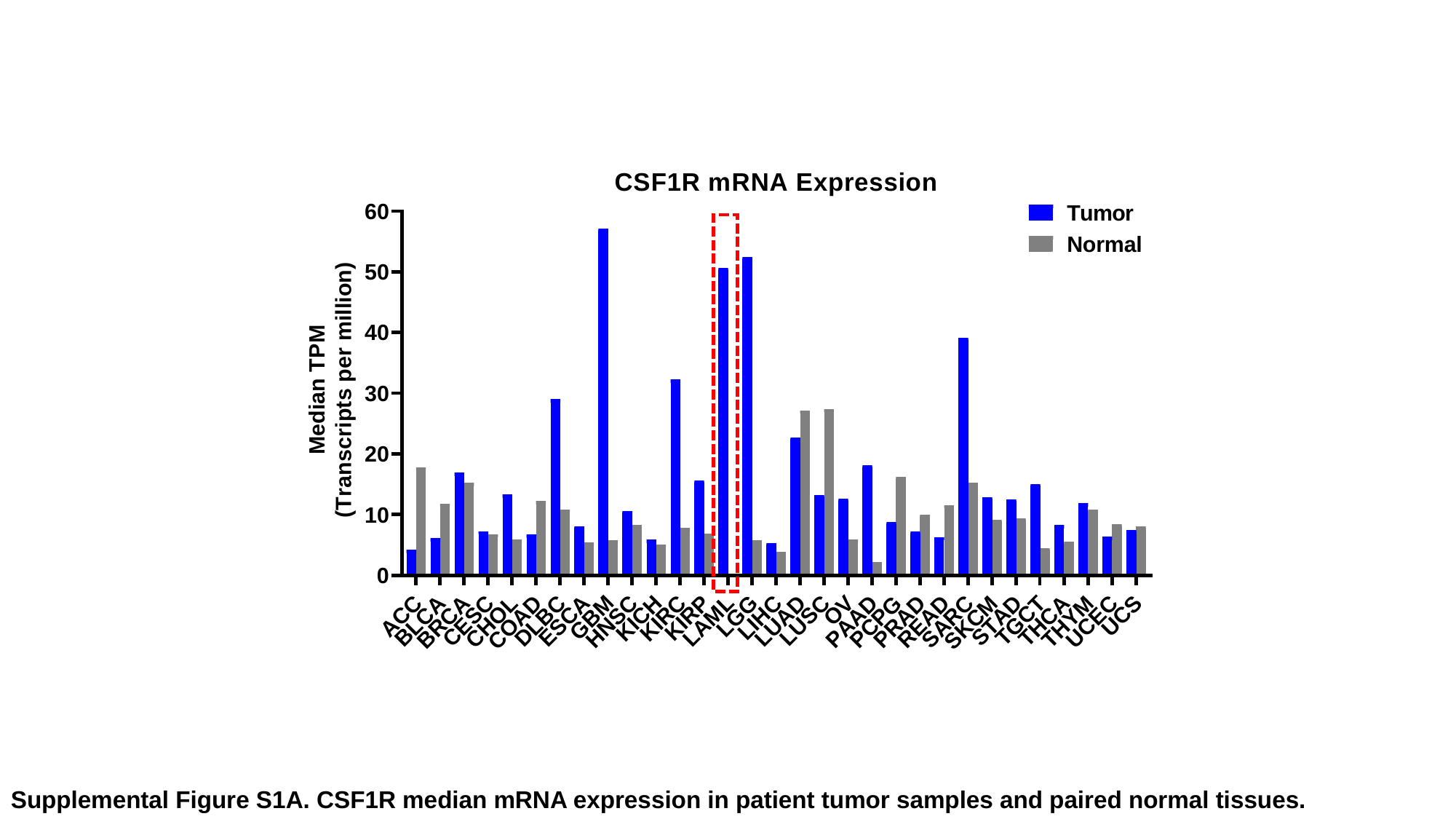

Supplemental Figure S1A. CSF1R median mRNA expression in patient tumor samples and paired normal tissues.

## Slide 3
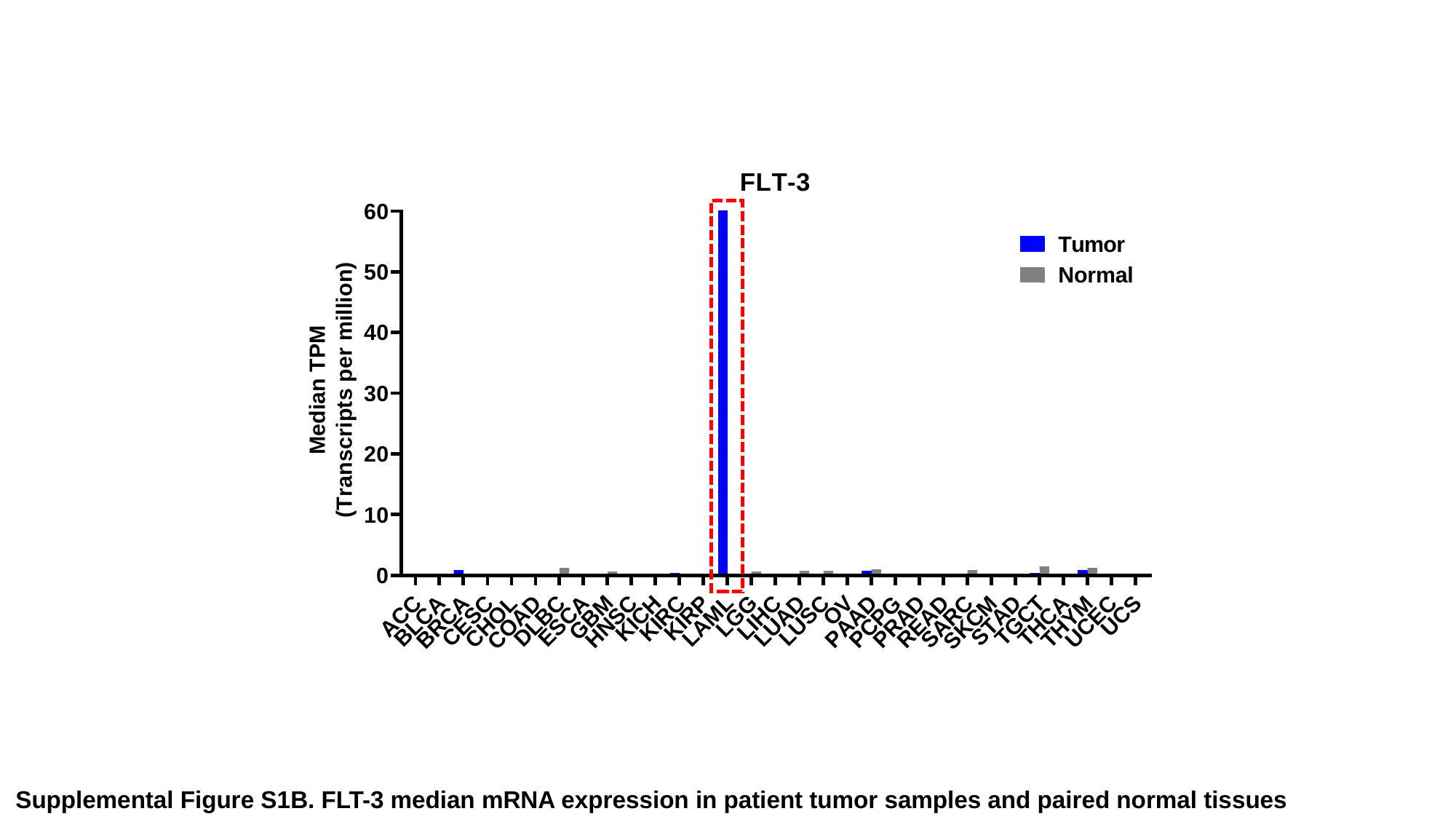

Supplemental Figure S1B. FLT-3 median mRNA expression in patient tumor samples and paired normal tissues

## Slide 4
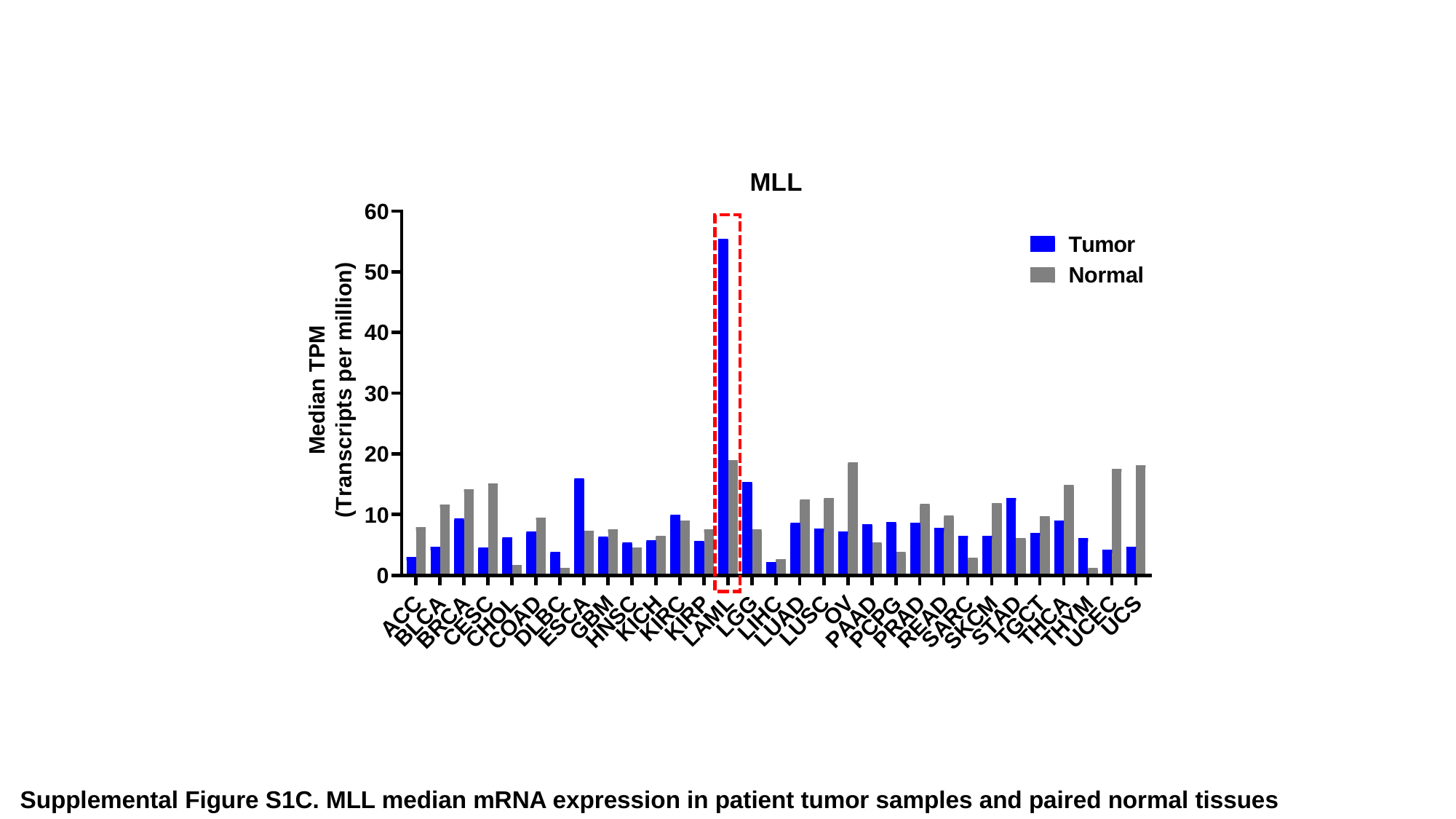

Supplemental Figure S1C. MLL median mRNA expression in patient tumor samples and paired normal tissues

## Slide 5
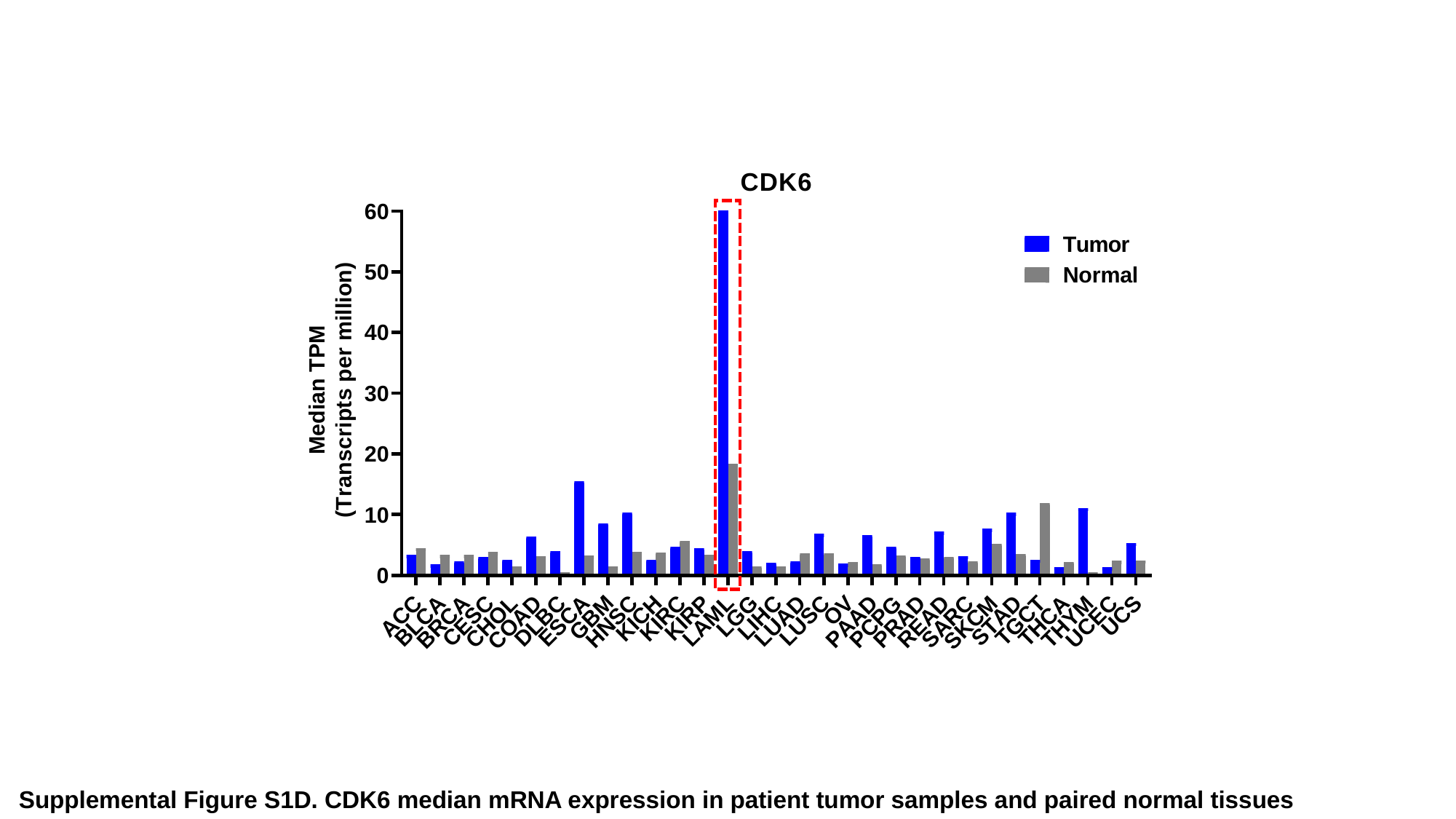

Supplemental Figure S1D. CDK6 median mRNA expression in patient tumor samples and paired normal tissues

## Slide 6
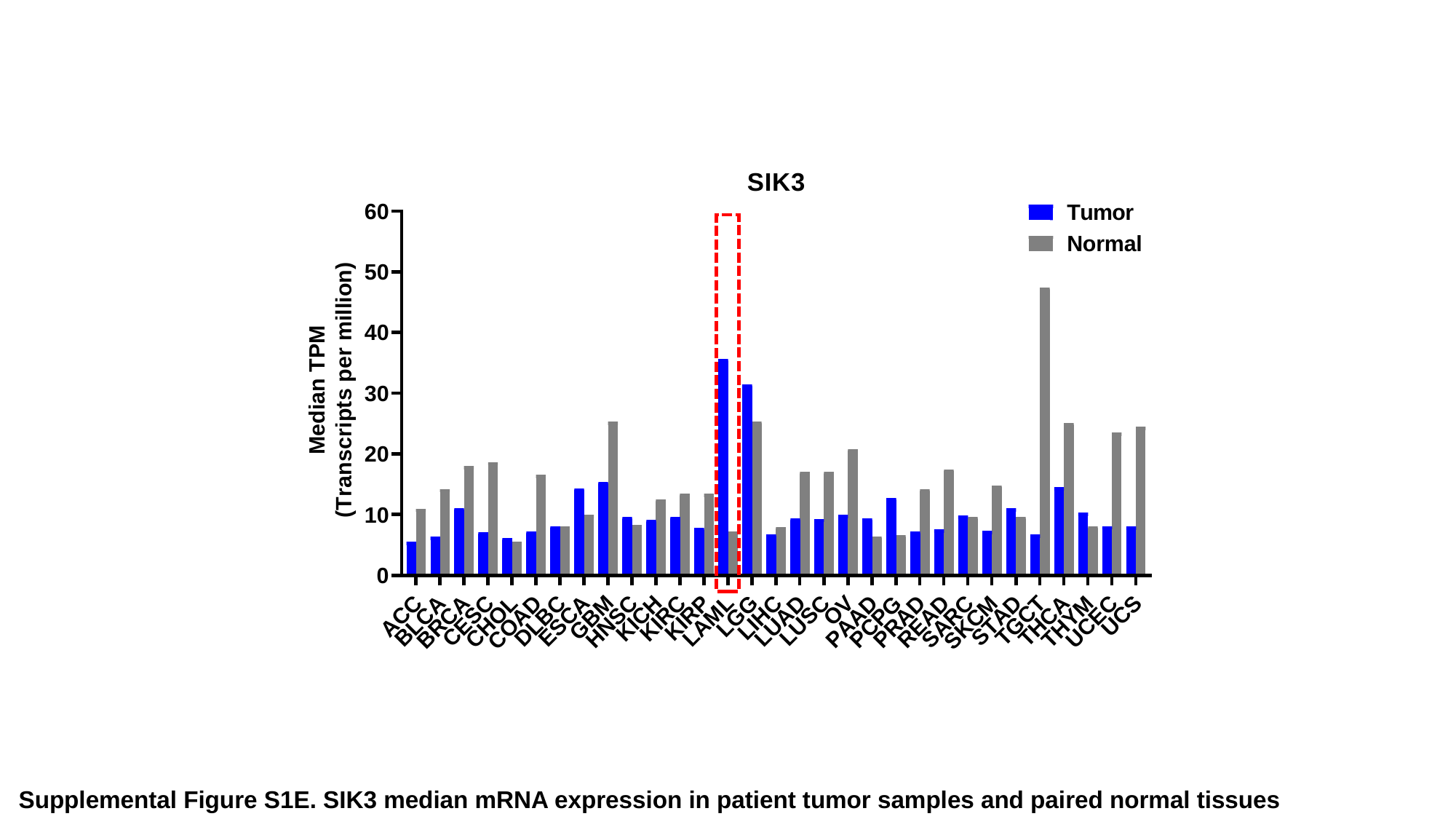

Supplemental Figure S1E. SIK3 median mRNA expression in patient tumor samples and paired normal tissues

## Slide 7
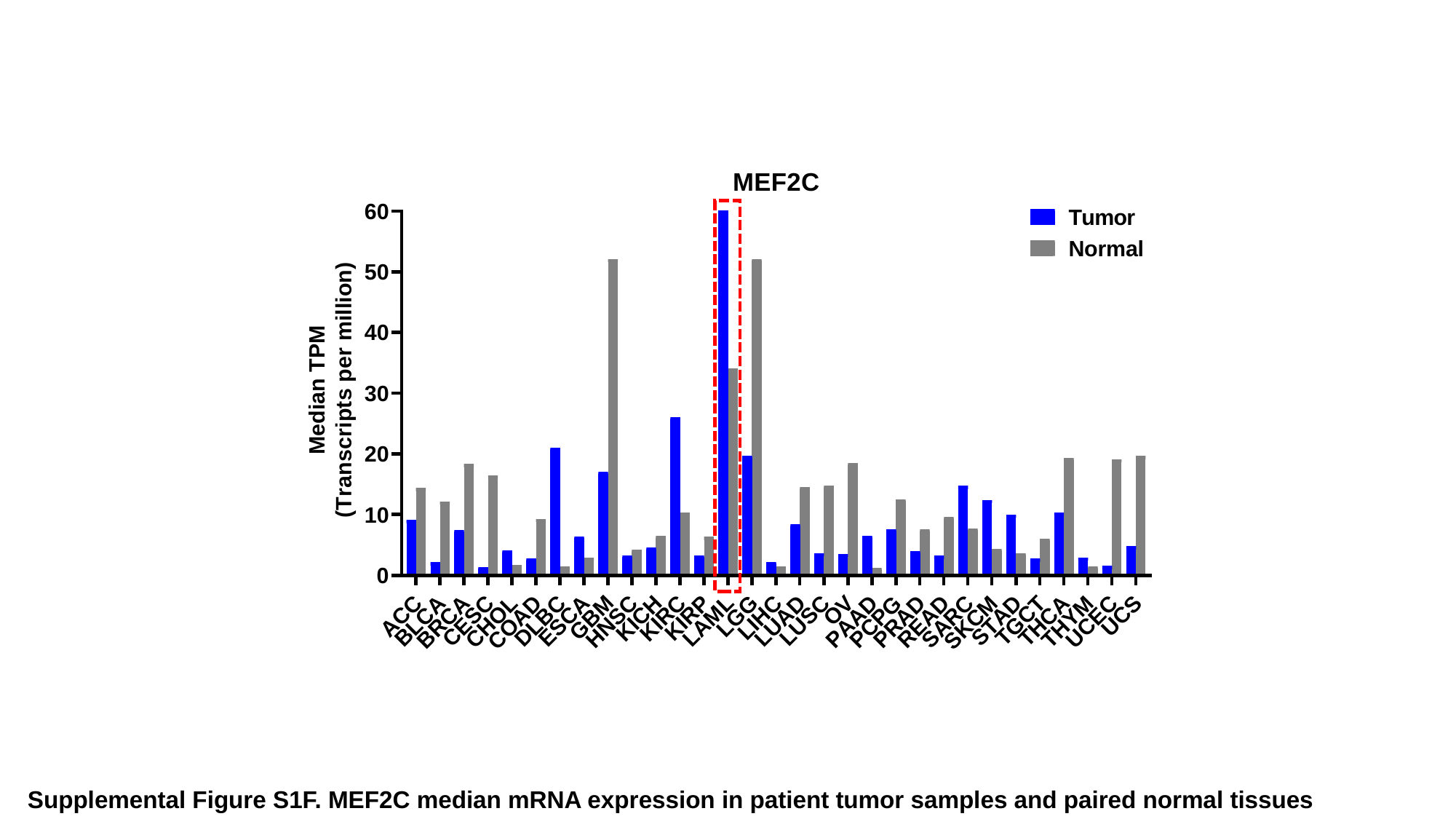

Supplemental Figure S1F. MEF2C median mRNA expression in patient tumor samples and paired normal tissues

## Slide 8
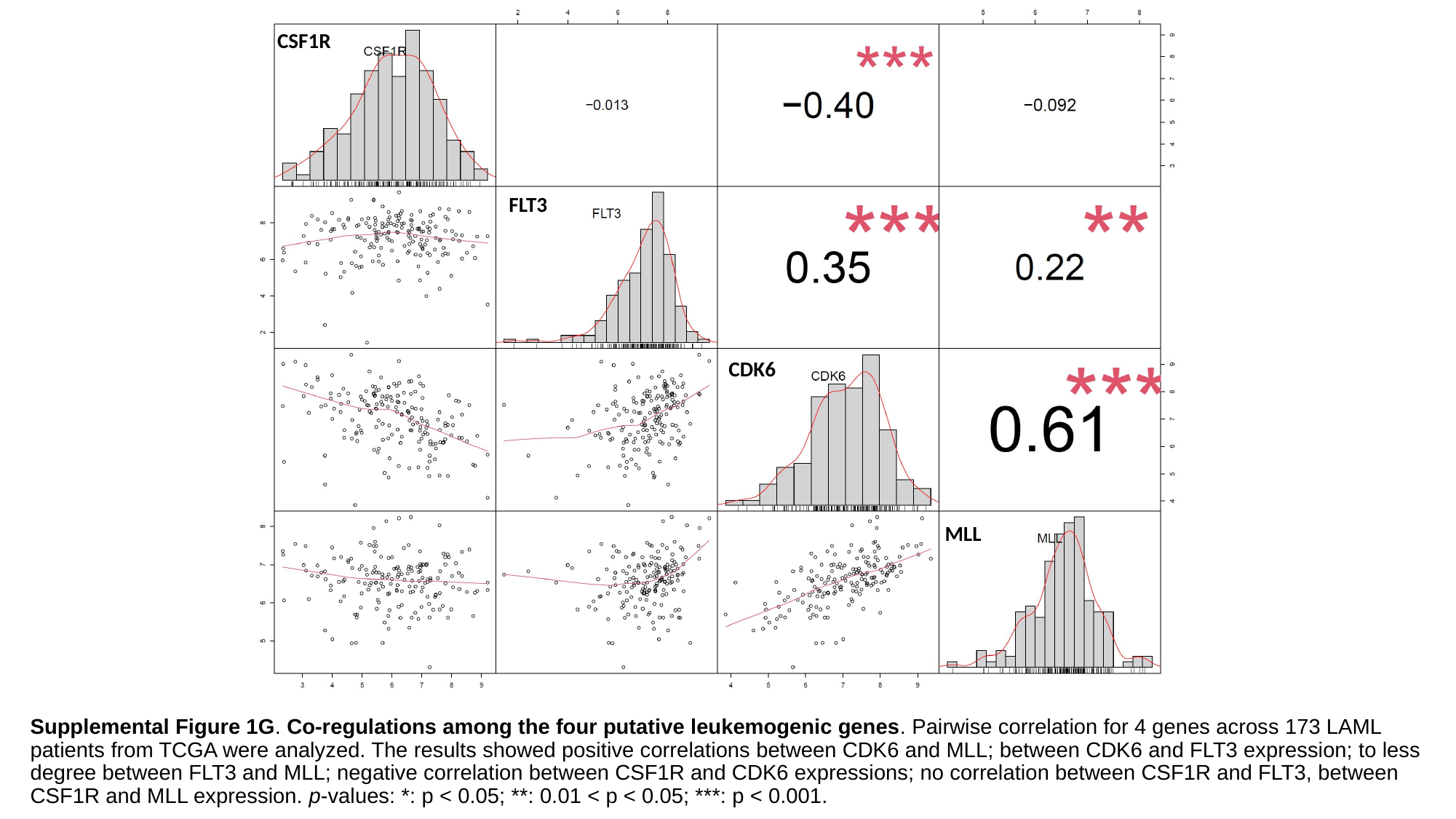

CSF1R
FLT3
CDK6
MLL
# Supplemental Figure 1G. Co-regulations among the four putative leukemogenic genes. Pairwise correlation for 4 genes across 173 LAML patients from TCGA were analyzed. The results showed positive correlations between CDK6 and MLL; between CDK6 and FLT3 expression; to less degree between FLT3 and MLL; negative correlation between CSF1R and CDK6 expressions; no correlation between CSF1R and FLT3, between CSF1R and MLL expression. p-values: *: p < 0.05; **: 0.01 < p < 0.05; ***: p < 0.001.

## Slide 9
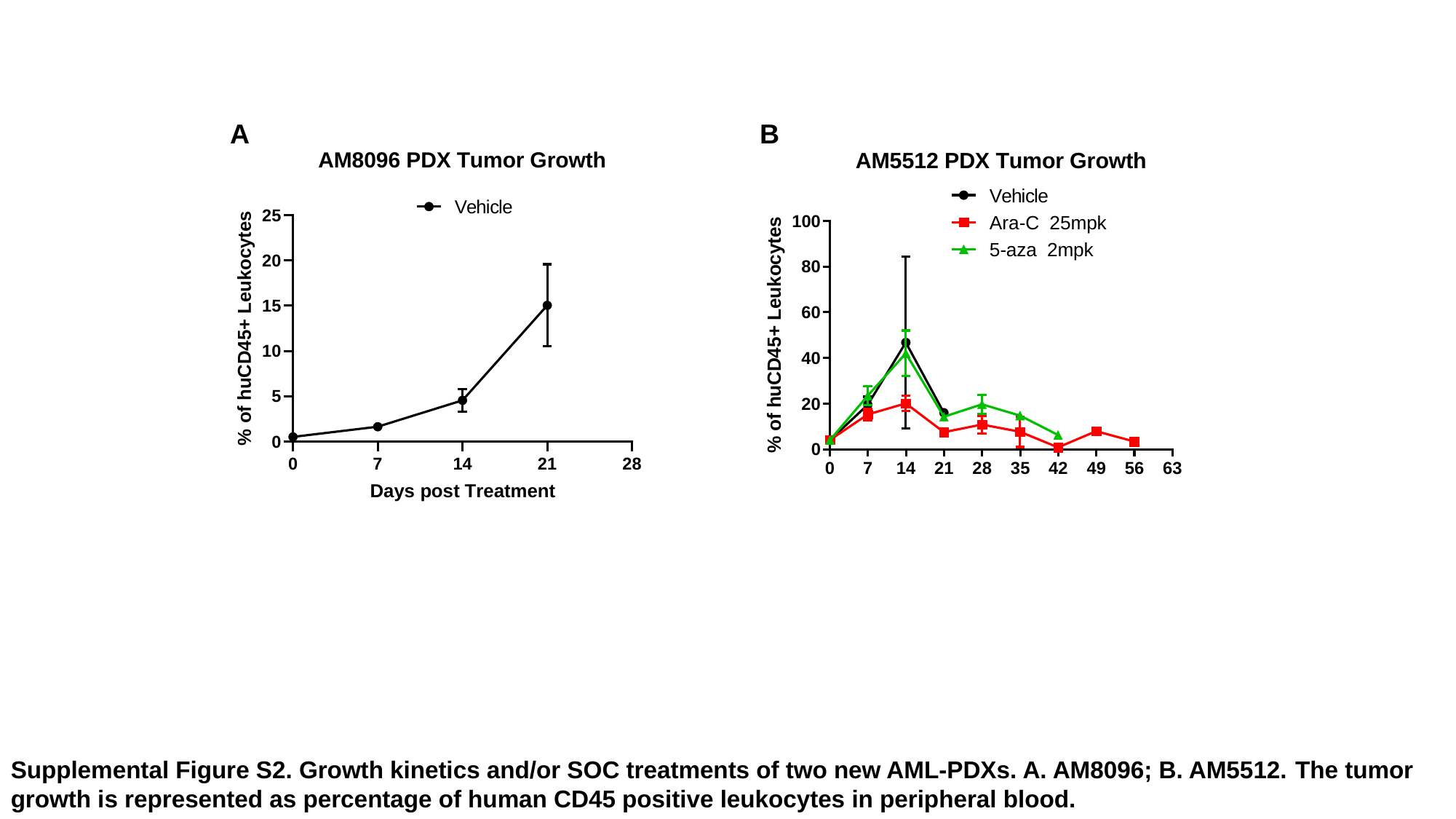

A
B
Supplemental Figure S2. Growth kinetics and/or SOC treatments of two new AML-PDXs. A. AM8096; B. AM5512. The tumor growth is represented as percentage of human CD45 positive leukocytes in peripheral blood.

## Slide 10
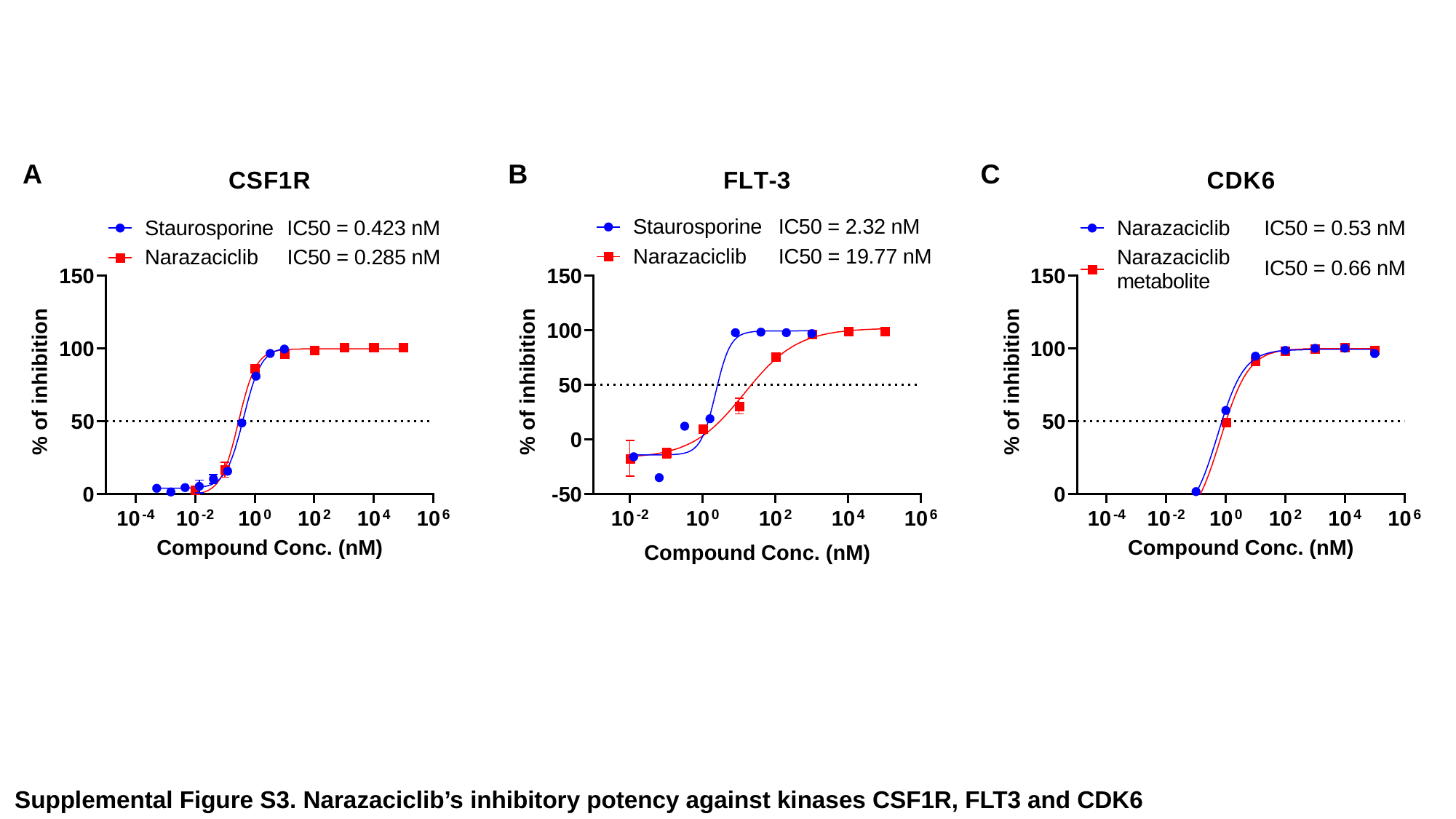

A
B
C
Supplemental Figure S3. Narazaciclib’s inhibitory potency against kinases CSF1R, FLT3 and CDK6

## Slide 11
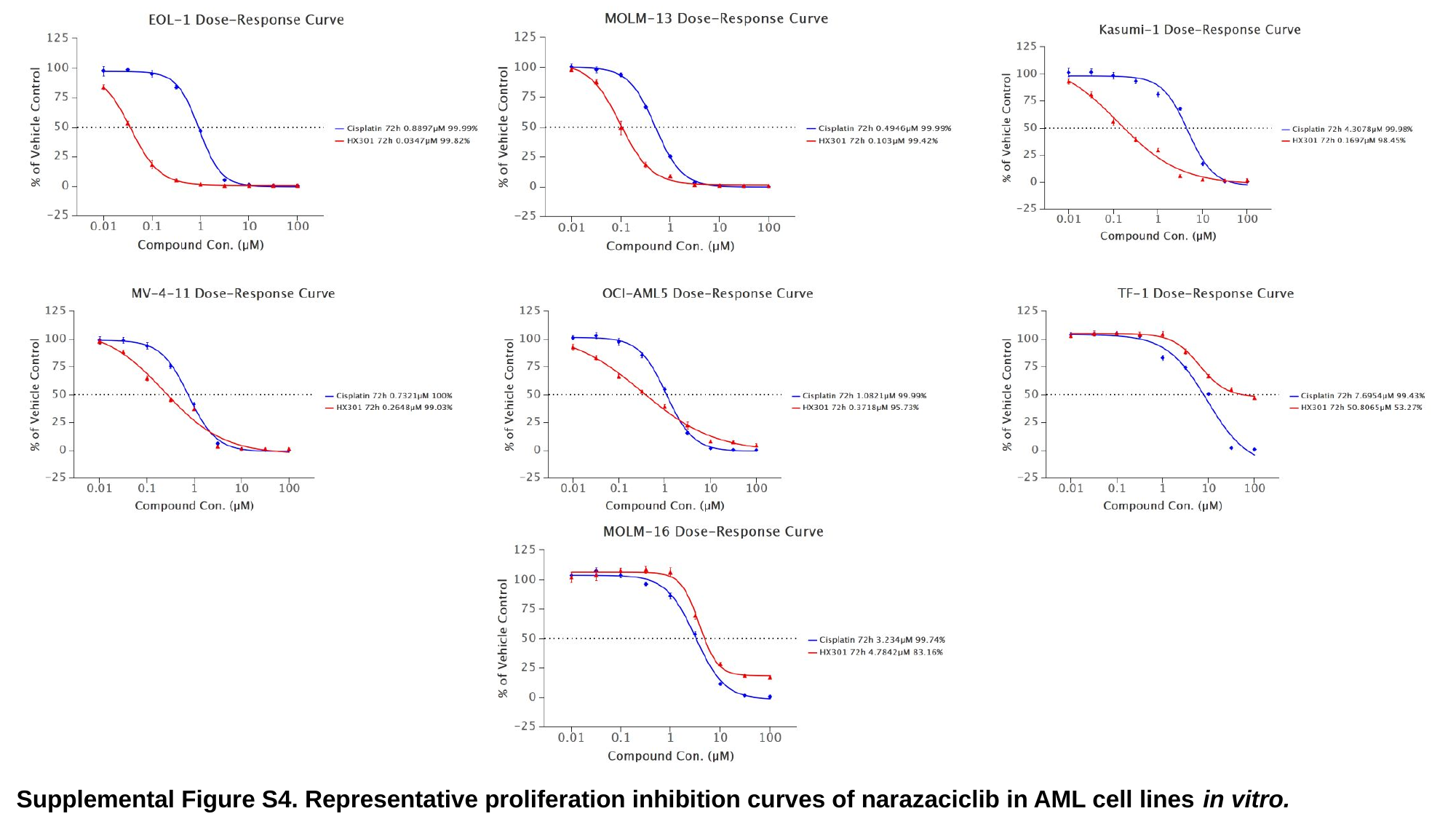

Supplemental Figure S4. Representative proliferation inhibition curves of narazaciclib in AML cell lines in vitro.

## Slide 12
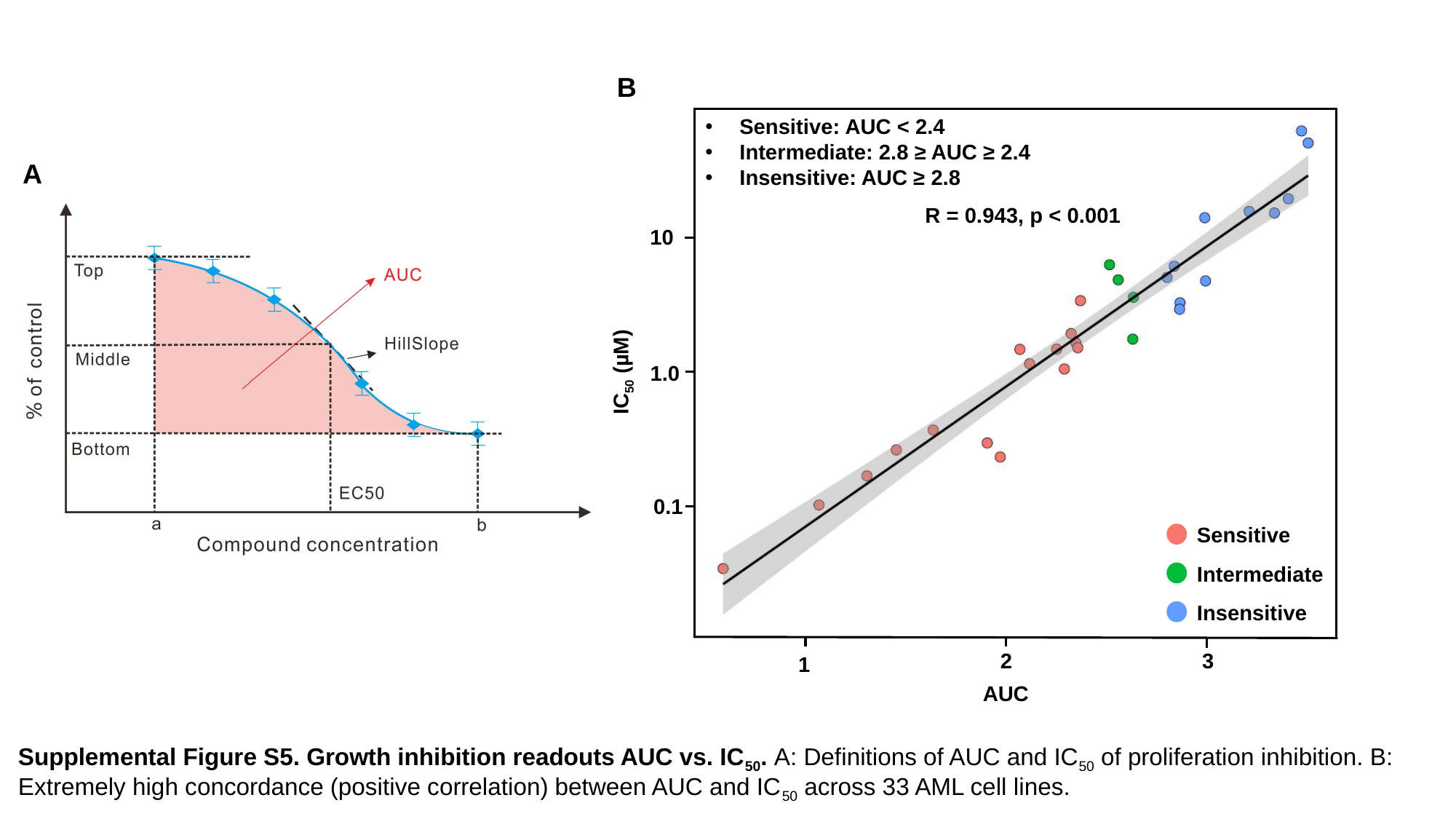

B
Sensitive: AUC < 2.4
Intermediate: 2.8 ≥ AUC ≥ 2.4
Insensitive: AUC ≥ 2.8
R = 0.943, p < 0.001
10
IC50 (µM)
1.0
0.1
Sensitive
Intermediate
Insensitive
2
3
1
AUC
A
# Supplemental Figure S5. Growth inhibition readouts AUC vs. IC50. A: Definitions of AUC and IC50 of proliferation inhibition. B: Extremely high concordance (positive correlation) between AUC and IC50 across 33 AML cell lines.

## Slide 13
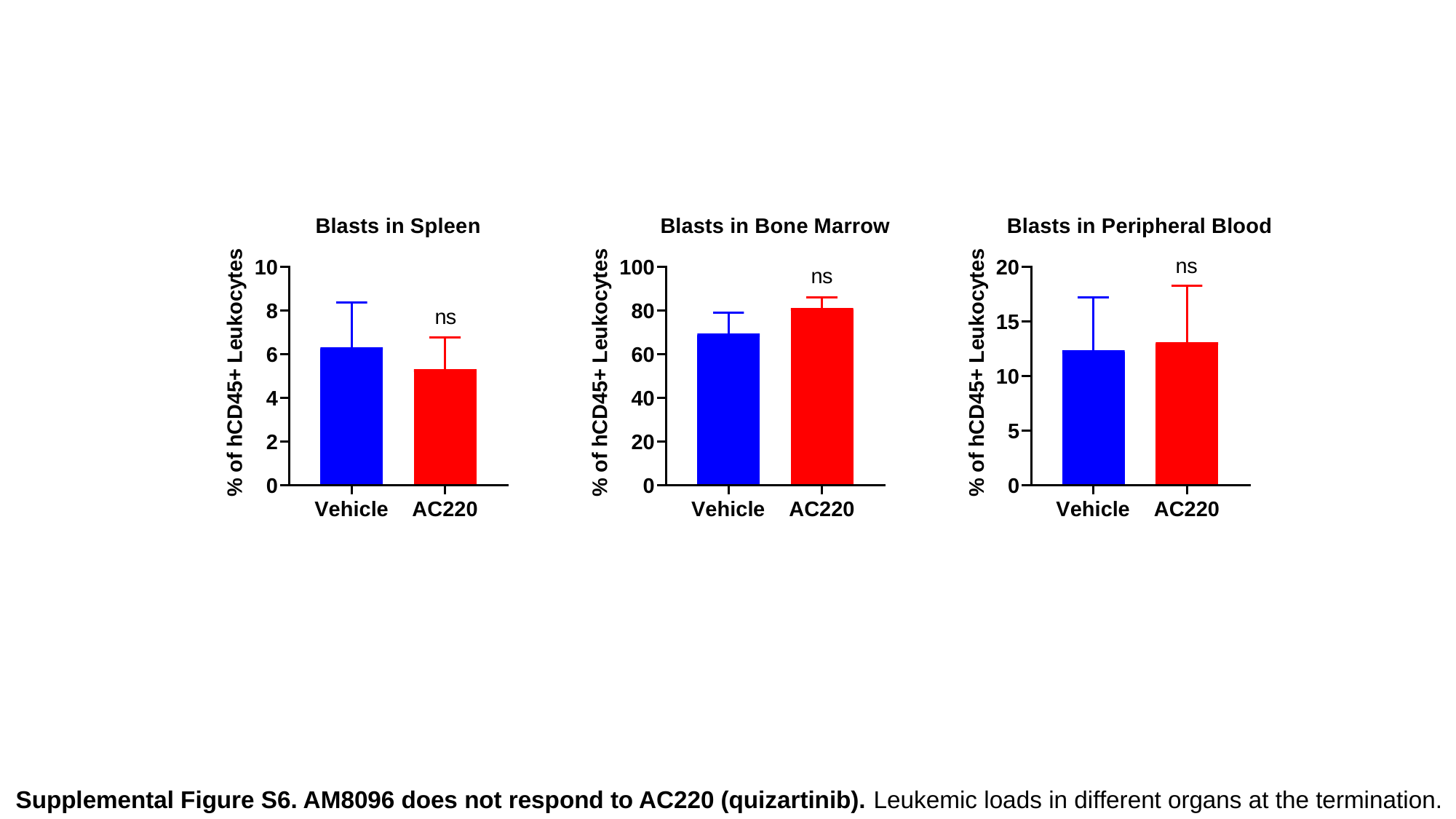

Supplemental Figure S6. AM8096 does not respond to AC220 (quizartinib). Leukemic loads in different organs at the termination.

## Slide 14
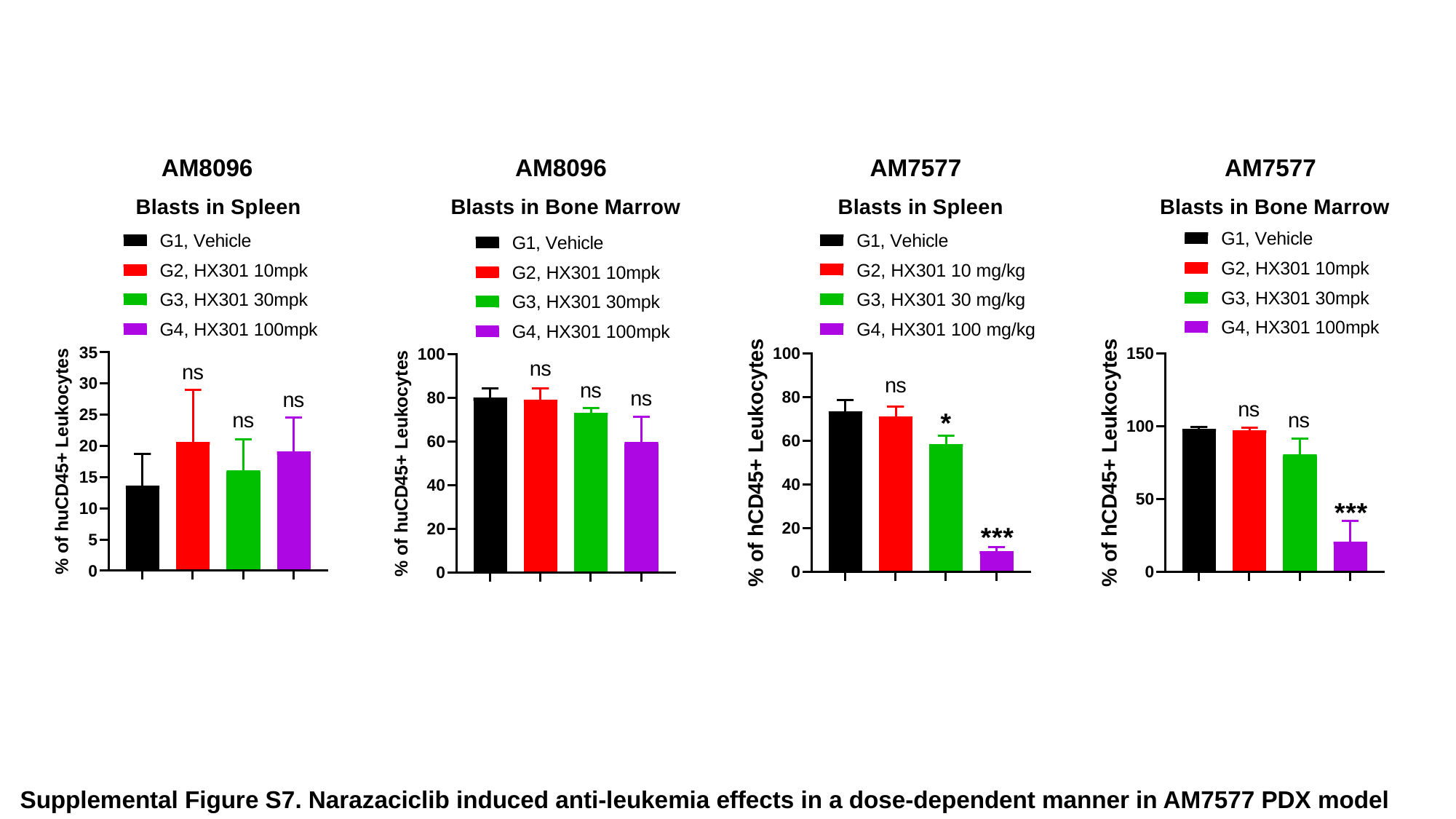

AM7577
AM8096
AM8096
AM7577
Supplemental Figure S7. Narazaciclib induced anti-leukemia effects in a dose-dependent manner in AM7577 PDX model

## Slide 15
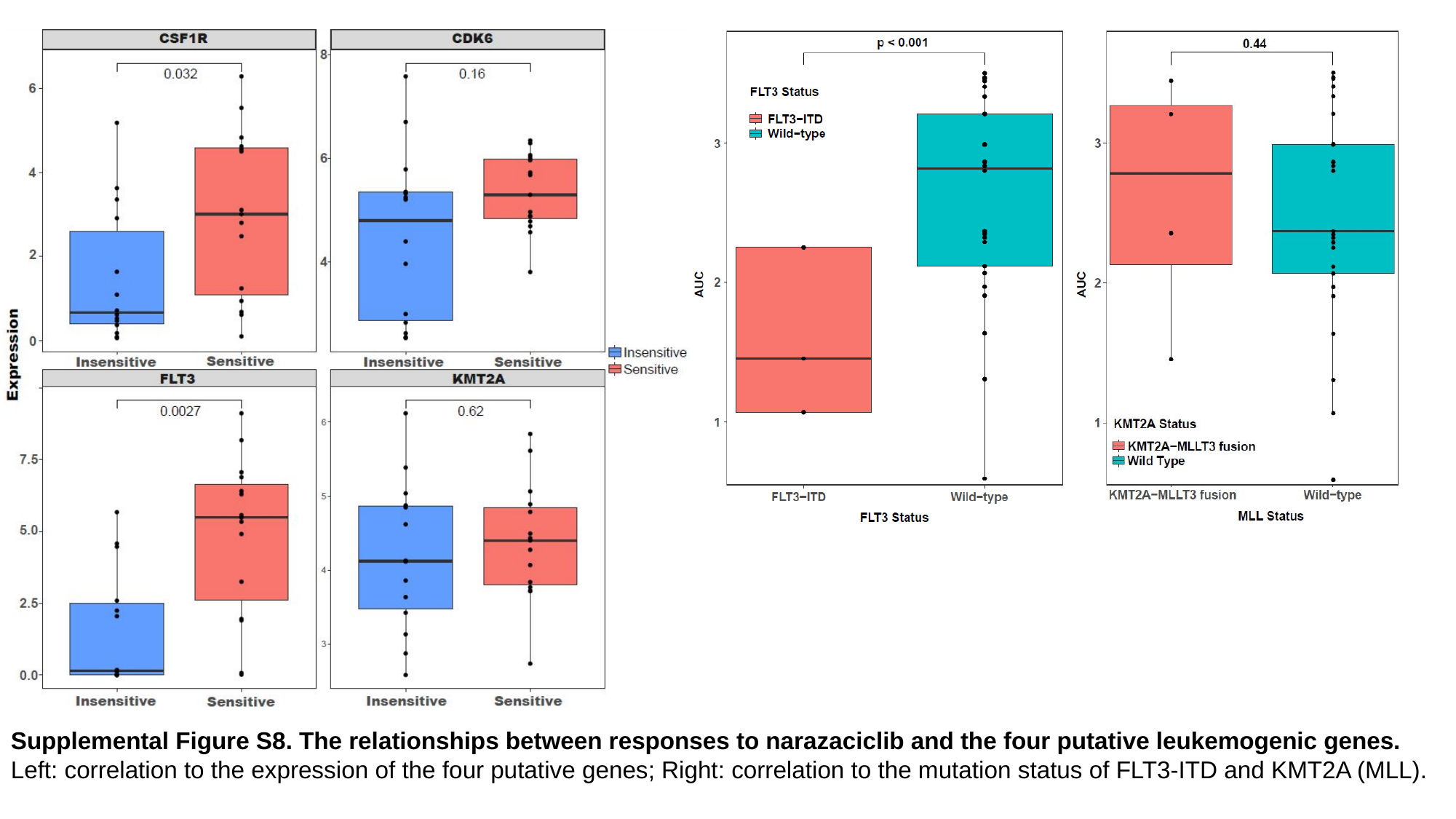

Supplemental Figure S8. The relationships between responses to narazaciclib and the four putative leukemogenic genes. Left: correlation to the expression of the four putative genes; Right: correlation to the mutation status of FLT3-ITD and KMT2A (MLL).
