# Supplementary material for: Narazaciclib, a novel multi-kinase inhibitor with potent activity against CSF1R, FLT3 and CDK6, shows strong anti-AML activity in defined preclinical models
Source: Sci Rep. 2024 Apr 19;14:9032. doi: 10.1038/s41598-024-59650-y (PMC11031590; doi:10.1038/s41598-024-59650-y)
Supplement: Supplementary file 2 — Supplementary Tables. [file 41598_2024_59650_MOESM2_ESM.docx]

**Supplemental Table S1. Patient information related to four AML-PDXs tested *in vivo***

| **ID** | **Subtype** | **Ethnicity** | **Sex** | **Age** | **Primary Blood Routine** | **Phenotypes** | **Genotypes** | **Cytogenetics** |
| --- | --- | --- | --- | --- | --- | --- | --- | --- |
| AM5512 | M7 | Asian | M | 15 | WBC: 39 x 10^9^/L, HB: 71 g/L,  PLT: 19 x 10^9^/L, Abnormal: 38%, | Positive for CD33,CD36,CD9,CD38, HLA-DR;  partially express:CD7,CD4,CD71,CD61, CD41,CD11b;  no express: CD2,CD3 | NA | NA |
| AM7577 | M5 | Asian | M | 69 | WBC: 38.9 x 10^9^/L, HB: 74.2 g/L,  PLT: 163 x 10^9^/L, Abnormal: 79.20%, Naive/nuclear: 11.47%.  Classification of blood: 63% | Positive for CD13/CD33/HLA-DR/CD117/CD38/CD71,  partially express MPO/CD15/CD19/CD7, not express CD34/CD10/CD20/CD79a/  CD3/CD5/CD11b/CD14/CD56/GlyA | CEBP-2(+ins c)/FLT3^+^/DNMT3A+/IDH2(R140Q)/NPM1 mutation | NA |
| AM8096 | M2 | Asian | M | 21 | WBC: 20 x 10^9^/L, blast cells 70%. | NA | RUNX1-RUNX1T1 fusion, CEBPA mutation | Normal Karyotype |
| AM7407 | M4 | Asian | M | 60 | WBC: 31.79 x 10^9^/L, HB: 58 g/L,  PLT: 122 x 10^9^/L | abnormal cells:20.25%, positive for CD13, CD33, CD34, HLA-DR, CD14, CD64, CD117 | KMT2A rearrangement | NA |

**Supplemental Table S2. Genetic and cytogenetic information of all tested AML cell lines**

| **Cell Line** | **FAB Classification** | **Genetic Features and Abnormalities** |
| --- | --- | --- |
| **EOL-1** | Eosinophilic | WT FLT-3, FIP1L1-PDGFRA fusion, KMT2A rearrangement |
| **MOLM-13** | M5a | FLT3-ITD, KMT2A rearrangement, *CEBPA* mutation |
| **KASUMI-1** | M2 | WT FLT-3, t(8;21), *RUNX1-RUNX1T1* fusion, trisomy of chromosome 10, monosomy of chromosome 13, *TP53* mutation, *c-KIT* mutation |
| **SKNO-1** | M2 | WT FLT-3, t(8;21) (q22;q22), *RUNX1/RUNX1T1* fusion, monosomy of chromosome 17 |
| **MV-4-11** | M5 | FLT3-ITD, KMT2A-MLL3 fusion, t(4;11) (q21;q23), KMT2A/MLLT2 (AF4) |
| **MONOMAC-1** | M5 | WT FLT-3, KMT2A-MLLT3 (AF9) fusion |
| **OCI-AML-5** | M4 | High level of WT FLT-3, *CEBPA* mutation, *BCORL1* mutation |
| **KG-1A** | M1 | WT FLT-3, FGFR1 fusions, *TP53* mutation |
| **OCI-AML-2** | M4 | WT FLT-3, *DNMT3A* R635W mutation, KMT2A rearrangement |
| **HNT34** | M4 | WT FLT-3, BCR-ABL1 fusion |
| **KASUMI-6** | M2 | FLT-3-ITD, trisomy of chromosome 10, *CEBPA* mutation, KMT2A rearrangement |
| **MONOMAC-6** | M5 | WT FLT-3, KMT2A-MLL3 fusion |
| **CMK** | M7 | WT FLT-3, der(17)t(11:17), *JAK2 V617F* mutation, |
| **KG-1** | M1 | WT FLT-3, *TP53* mutation, FGFR1 fusions |
| **SKM-1** | MDS | High level of WT FLT-3, del(9)(q13;q22), *ASXL1* mutations, *KRAS* mutation, *TP53* mutation |
| **OCI-AML-3** | M4 | WT FLT-3, *DNMT3A* R882 mutations, *NPM1* mutations |
| **NB4** | M3 | WT FLT-3, t(15;17) PML-RARA fusion, *KRAS* mutation, *TP53* mutation |
| **PL21** | M3 | WT FLT-3 and FLT-3-ITD, *KRAS* mutation, *TP53* mutation |
| **ML2** | M4 | WT FLT-3, KMT2A-MLLT4 (AF6) fusion, *KRAS* mutation |
| **MOLM-16** | M0 | High level of WT FLT-3, *TP53* mutation, *CEBPA* mutation |
| **M07E** | M7 | WT FLT-3, ETO2-GLIS2 fusion |
| **CMK-11-5** | M7 | WT FLT-3, TP53-FXR2 fusion, *TP53* mutation |
| **HL-60** | M2 | WT FLT-3, *TP53* loss, deletions of chromosomes 5, 8, and X, CDKN2A mutation |
| **AML-193** | M5 | WT FLT-3, *BCORL1* mutation, *TP53* mutation |
| **PLB-985** | M2 | WT FLT-3, *TP53* loss, CDKN2A mutation |
| **HEL** | M6 | WT FLT-3, *JAK2 V617F* mutation, *TP53* mutation |
| **HEL-92.1.7** | M6 | WT FLT-3, *JAK2 V617F* mutation, *TP53* mutation |
| **OCI-M1** | M6 | WT FLT-3, *TP53* mutation |
| **TF-1** | M6 | WT FLT-3, homogenous chromosomal abnormality (54, X), |
| **UT7** | M7 | WT FLT-3, CBFA2T3-ABHD12 fusion, *TP53* mutation |
| **NOMO-1** | M5a | WT FLT-3, KMT2A-MLL3 fusion, *CEBPA* mutation, *KRAS* mutation, *TP53* mutation |
| **OCI-M2** | M6 | WT FLT3, RUNX1-TSPEAR fusion, *TP53* mutation |
| **THP-1** | M5 | WT FLT-3, KMT2A/MLLT3(AF9) fusion, PICALM/MLLT10 (AF10), *TP53* mutation |

**Supplemental Table S3. Abbreviations for different tumor types.**

| ACC | Adrenocortical carcinoma |
| --- | --- |
| BLCA | Bladder Urothelial carcinoma |
| BRCA | Breast invasive carcinoma |
| CESC | Cervical squamous cell carcinoma and endocervical adenocarcinoma |
| CHOL | Cholangial carcinoma |
| COAD | Colon adenocarcinoma |
| DLBC | Lymphoid Neoplasm Diffuse Large B-cell Lymphoma |
| ESCA | Esophageal carcinoma |
| GBM | Glioblastoma multiforme |
| HNSC | Head and Neck squamous cell carcinoma |
| KICH | Kidney Chromophobe |
| KIRC | Kidney renal clear cell carcinoma |
| KIRP | Kidney renal papillary cell carcinoma |
| LAML | Acute Myeloid Leukemia |
| LGG | Brain Lower Grade Glioma |
| LIHC | Liver hepatocellular carcinoma |
| LUAD | Lung adenocarcinoma |
| LUSC | Lung squamous cell carcinoma |
| MESO | Mesothelioma |
| OV | Ovarian serous cystadenocarcinoma |
| PAAD | Pancreatic adenocarcinoma |
| PCPG | Pheochromocytoma and Paraganglioma |
| PRAD | Prostate adenocarcinoma |
| READ | Rectum adenocarcinoma |
| SARC | Sarcoma |
| SKCM | Skin Cutaneous Melanoma |
| STAD | Stomach adenocarcinoma |
| TGCT | Testicular Germ Cell Tumors |
| THCA | Thyroid carcinoma |
| THYM | Thymoma |
| UCEC | Uterine Corpus Endometrial Carcinoma |
| UCS | Uterine Carcinosarcoma |
| UVM | Uveal Melanoma |

**Supplemental Table S4. IC_50_ of Narazaciclib against additional kinases**

| **Kinase** | **Narazaciclib (µM)** | **Narazaciclib metabolite (µM)** |
| --- | --- | --- |
| ABL-1 | 0.038 | 0.028 |
| C-Kit | 0.009 | 0.012 |
| CDK9 | 0.028 | 0.025 |
| FGFR1 | 0.038 | 0.050 |
| FGFR2 | 0.020 | 0.028 |
| FGFR3 | 0.187 | 0.104 |
| FGFR4 | 2.745 | 2.697 |
| PDGFRα | 0.104 | 0.040 |
| PDGFRβ | 0.010 | 0.017 |
| RET | 0.016 | 0.065 |
| SIK1 | 0.034 | 0.041 |
| SIK2 | 0.002 | 0.003 |
| SIK3 | 0.024 | 0.027 |
